# Truncated PARP1 mediates ADP-ribosylation of RNA polymerase III for apoptosis

**DOI:** 10.1038/s41421-021-00355-1

**Published:** 2022-01-18

**Authors:** Qian Chen, Kai Ma, Xiuhua Liu, Shih-Hsun Chen, Peng Li, Yonghao Yu, Anthony K. L. Leung, Xiaochun Yu

**Affiliations:** 1 Department of Cancer Genetics and Epigenetics, Beckman Research Institute, City of Hope Medical Center, Duarte, CA USA; 2 College of Life Sciences, Hebei University, Baoding, Hebei China; 3 Institute of Biochemical Sciences, National Taiwan University, Taipei, Taiwan China; 4 Department of Biochemistry, University of Texas Southwestern Medical Center, Dallas, TX USA; 5 Department of Biochemistry and Molecular Biology, Bloomberg School of Public Health, Johns Hopkins University, Baltimore, MD USA; 6 Department of Molecular Biology and Genetics, School of Medicine, Johns Hopkins University, Baltimore, MD USA; 7 Department of Oncology, School of Medicine, Johns Hopkins University, Baltimore, MD USA; 8 Westlake Laboratory of Life Sciences and Biomedicine, Hangzhou, Zhejiang China; 9 School of Life Sciences, Westlake University, Hangzhou, Zhejiang China; 10 Institute of Basic Medical Sciences, Westlake Institute for Advanced Study, Hangzhou, Zhejiang China

**Keywords:** Apoptosis, PolyADP-ribosylation

## Abstract

Caspase-mediated cleavage of PARP1 is a surrogate marker for apoptosis. However, the biological significance of PARP1 cleavage during apoptosis is still unclear. Here, using unbiased protein affinity purification, we show that truncated PARP1 (tPARP1) recognizes the RNA polymerase III (Pol III) complex in the cytosol. tPARP1 mono-ADP-ribosylates RNA Pol III in vitro and mediates ADP-ribosylation of RNA Pol III during poly(dA-dT)-stimulated apoptosis in cells. tPARP1-mediated activation of RNA Pol III facilitates IFN-β production and apoptosis. In contrast, suppression of PARP1 or expressing the non-cleavable form of PARP1 impairs these molecular events. Taken together, these studies reveal a novel biological role of tPARP1 during cytosolic DNA-induced apoptosis.

## Introduction

Apoptosis plays a key role in maintaining homeostasis for the survival in multicellular organism, especially under pathophysiological stresses^1–3^. One typical example is the pathogenic infection-induced innate immune response, which is the first line of defense against infections and facilitates organism’s survival via eliciting apoptosis that eliminates the infected cells to prevent pathogen replication and propagation^4–7^.

During apoptosis, a group of highly conserved cysteinyl-aspartate proteases (caspases) are activated and promote apoptosis^8,9^. One prominent substrate of caspases is poly(ADP-ribose) polymerase 1 (PARP1)^10,11^. In fact, the cleavage of PARP1 is a hallmark of apoptosis^3,12,13^.

PARP1 is the founding member of PARP family and catalyzes poly(ADP-ribosyl)ation (also known as PARylation) on protein substrates^14–17^. It contains multiple domains, including the N-terminal zinc finger motifs, the BRCT domain, the WGR domain and the C-terminal catalytic domain^18,19^. The activation of PARP1 is mainly induced by DNA single-strand breaks or double-strand breaks, which are recognized by the zinc finger motifs as well as the WGR domain. Through an intramolecular conformational change, the catalytic site of PARP1 is exposed and recognizes the donor NAD^+^ for PARylation on protein substrates^20–25^. PARP1 is involved in multiple cellular processes, such as DNA damage repair and transcriptional regulation^26–31^. The major functions of PARP1 are dependent on its catalytic activity^27,32–35^.

However, during apoptosis, the first two zinc finger motifs of PARP1 are cleaved mainly by caspase 3 to generate two fragments with 24 and 89 kD, respectively^8,10,11,36^. The 89 kD truncated PARP1 (tPARP1) contains most domains and relocates from the nucleus to the cytoplasm, whereas the 24 kD N-terminal fragment has the nuclear localization sequence and remains in the nucleus^37–42^. Since the N-terminal fragment contains the first two zinc finger motifs, it may still recognize and occupy DNA ends, which acts as the dominant-negative form to suppress DNA end sensing and PARP1-mediated DNA damage repair during apoptosis^41^. However, the biological function of tPARP1 is still unclear. Interestingly, PARP1 is conserved during evolution. In some lower eukaryotes, PARP1 does not have the first two zinc finger motifs, which is similar to tPARP1 generated during apoptosis, suggesting that tPARP1 has its own biological functions^43–45^.

To elucidate the biological function of tPARP1, we performed unbiased protein affinity purification and unexpectedly found that tPARP1 recognizes RNA polymerase III (Pol III). Interestingly, Pol III plays a key role during the innate immune response via transcription of foreign DNA from invading pathogens, which stimulates interferon beta (IFN-β) production. Our results show that tPARP1 catalyzes ADP-ribosylation on Pol III, which facilitates IFN-β production and apoptosis. Collectively, our studies reveal a biological function of tPARP1 in apoptosis.

## Results

### Identification of Pol III complex as tPARP1-binding partners

**Fig. 1:**
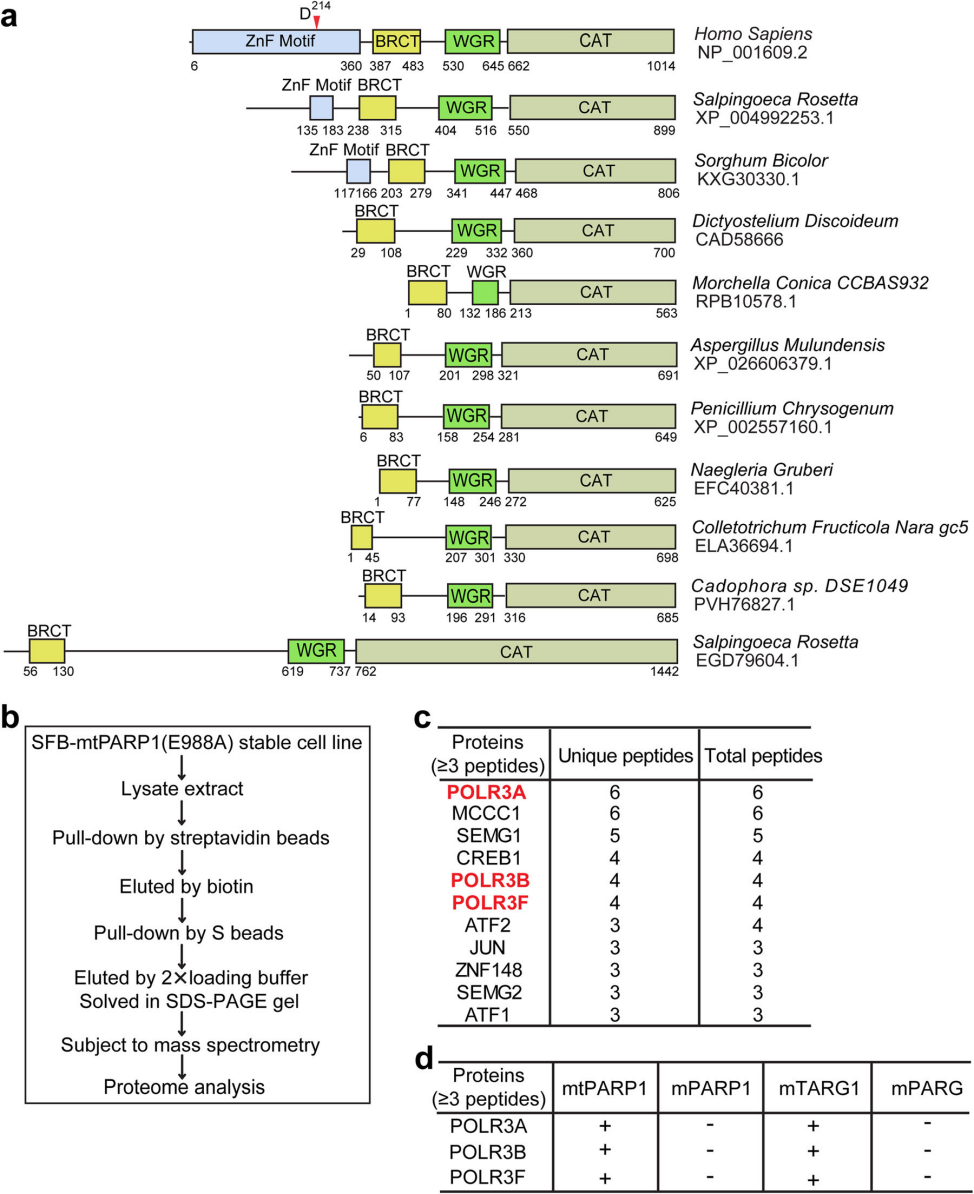
Identification of Pol III complex as tPARP1-binding partners. **a** The domain architecture of human PARP1 and its orthologs in a set of lower eukaryotes. Caspase 3 cleavage site on human PARP1 is at D214. **b** A schematic representation of the tandem affinity purification assay to identify mutant tPARP1-binding partners. **c** List of the top 11 unique proteins with more than two unique peptides identified from mutant tPARP1 (mtPARP1) purification using mass spectrometry. **d** Pol III complex was only identified in the protein affinity purification using mtPARP1 and mutant TARG1 (mTARG1) but not from that using mutant PARP1 (mPARP1) or mutant PARG (mPARG).

The three N-terminal zinc finger motifs of PARP1 are evolutionarily conserved in multicellular organism^43–45^. However, during apoptosis, human PARP1 is mainly cleaved by caspase 3 at D214^8,11^. As a result, the tPARP1 loses two N-terminal zinc finger motifs and only contains the third zinc finger motif, the BRCT domain, the WGR domain, and the C-terminal catalytic domain. Interestingly, when we explored the domain architecture of PARP1 in other organisms, we found that, similar to human tPARP1, PARP1 orthologs in several lower organisms do not have the two N-terminal zinc fingers or even lack the third zinc finger (ZnF3) motif (Fig. 1a). This indicates that, even without the first two zinc finger motifs, tPARP1 may still catalyze ADP-ribosylation and play an important role in certain biological processes.

To reveal the biological function of tPARP1, we performed tandem affinity purification and searched for the possible substrates. Since the interaction between tPARP1 and its substrate(s) is very transient, we mutated the catalytic E988 residue into Ala to abolish the potential catalytic activity of tPARP1 and expected that mutant tPARP1 (mtPARP1) was able to trap the substrate(s). The SFB-tagged mtPARP1 was stably expressed in PARP1-deficient 293T cells, and the soluble fraction was isolated followed by tandem affinity purifications. The interacting proteins were analyzed by mass spectrometry (Fig. 1b, c and Supplementary Table S1). Usually, ADP-ribosylation occurs transiently because of the presence of potent enzymes that can remove ADP-riboses (ADPrs). Among the enzymes, PARG mediates the digestion of the glycosidic bond between two ADPrs, whereas TARG1 cuts the last ADPr residue from the Asp or Glu acceptor on the protein substrates^46,47^. Alternatively, TARG1 is able to remove mono-ADP-ribosylation (MARylation)^17,48^. Thus, if tPARP1 is able to catalyze ADP-ribosylation, the substrate(s) will be the common substrate(s) of PARG or TARG1 as well. Therefore, we mutated the key catalytic residue in either PARG (E756 to Ala, mPARG) or TARG1 (D125 to Ala, mTARG1). Using the similar strategy, we expected to use mPARG or mTARG1 to trap the common substrate(s) with mtPARP1. The proteins interacting with mPARG or mTARG1 were also unbiasedly purified from the 293T cells stably expressing mPARG or mTARG1 using the same tandem affinity purification strategy. With mass spectrometry analysis, interestingly, we found that POLR3A, POLR3B, and POLR3F, the three subunits of RNA polymerase III (Pol III) complex, were the common interacting proteins with both mtPARP1 and mTARG1 (Fig. 1d and Supplementary Tables S1 and S2). The mass spectrometry proteomics data have been deposited to the ProteomeXchange Consortium via the PRIDE partner repository with the data set identifier PXD018691. Collectively, these results suggest that mtPARP1 may recognize the Pol III complex.

### The BRCT domain of tPARP1 interacts with the Pol III complex

**Fig. 2:**
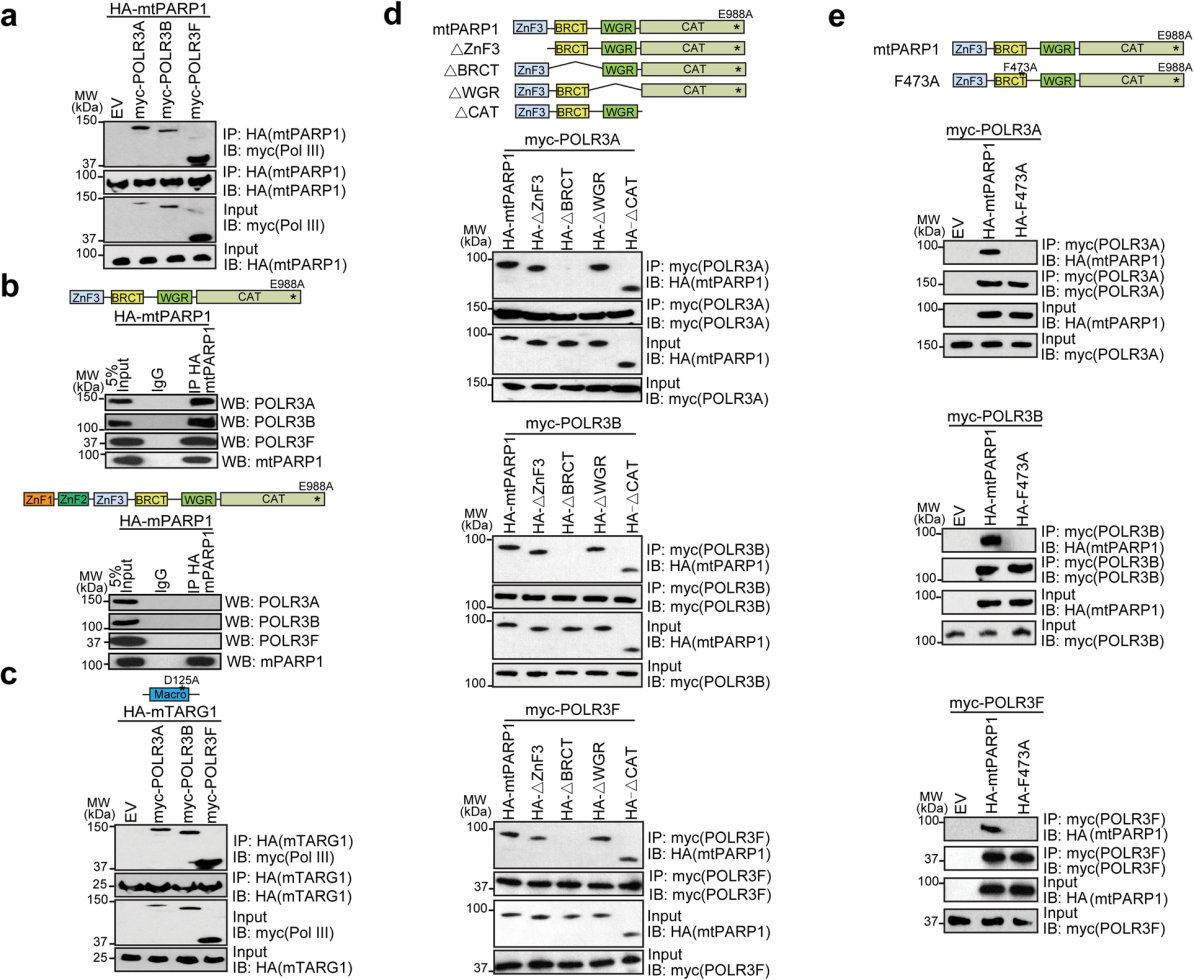
The BRCT domain of tPARP1 interacts with the Pol III complex. **a** U2OS-PARP1 knockout cells were transfected with the myc-empty vector (EV), myc-POLR3A, myc-POLR3B, or myc-POLR3F together with HA-mtPARP1, followed by IP and western blotting with the indicated antibodies. **b** mtPARP1 or full-length mPARP1 was ectopically expressed in U2OS-PARP1 knockout cells. IP was performed with IgG as a negative control. **c** U2OS-PARP1 knockout cells were transfected with the myc-empty vector (EV), myc-POLR3A, myc-POLR3B, or myc-POLR3F together with HA-mTARG1, followed by IP and western blotting with the indicated antibodies. **d** U2OS-PARP1 knockout cells were transfected with the HA-tagged truncation mutants of mtPARP1 together with myc-POLR3A (upper panel), myc-POLR3B (middle panel), or myc-POLR3F (lower panel). **e** U2OS-PARP1 knockout cells were transfected with the indicated combinations of HA-tagged mtPARP1 or mtPARP1 F473A mutant together with myc-POLR3A (upper panel), myc-POLR3B (middle panel), or myc-POLR3F (lower panel).

To validate the proteomics results, we expressed hemagglutinin (HA)-tagged mtPARP1 and myc-tagged three Pol III subunits in PARP1-deficient cells. We performed co-immunoprecipitation (co-IP) assays and found that mtPARP1 interacted with POLR3A, POLR3B, and POLR3F (Fig. 2a). Moreover, we expressed HA-tagged mtPARP1 and full-length PARP1 with the E988A mutation (mPARP1) in PARP1-deficient cells and found that only mtPARP1 but not the mPARP1 interacted with POLR3A, POLR3B, and POLR3F (Fig. 2b). Using co-IP assays, we confirmed that these Pol III subunits also interacted with mTARG1 in cells (Fig. 2c).

To map the interaction domain, we generated a series of internal truncation mutants of mtPARP1 and found that lacking BRCT domain suppressed the interactions with POLR3A, POLR3B, or POLR3F, whereas other domains were not required for the interactions (Fig. 2d). Interestingly, the BRCT domain is an evolutionarily conserved domain mediating protein–protein interaction. We mutated the key residue F473 present in the BRCT domain into Ala, to abolish the tertiary structure. The F473A mutation disrupted the interaction between mtPARP1 and the Pol III subunits (Fig. 2e). Taken together, the BRCT domain of tPARP1 recognizes the Pol III complex.

### tPARP1 recognizes the Pol III complex during apoptosis

Since tPARP1 does not exist until caspase 3 cuts the full-length PARP1 during apoptosis, we asked whether the interactions between mtPARP1 and the Pol III complex occur during apoptosis. In addition, since mtPARP1 translocates to the cytoplasm, it also indicates that the interaction may only occur in the cytoplasm during apoptosis. Interestingly, the Pol III complex resides in cytoplasm and senses double-stranded DNA (dsDNA) from pathogens during pathogen invasion^49,50^. This pathogen infection-induced stress response allows the Pol III complex to recognize foreign dsDNA and transcribes double-stranded RNA (dsRNA), which triggers innate immune response, such as IFN-β expression for apoptosis. Thus, we wondered whether mtPARP1 interacts with the Pol III complex in cytoplasm during apoptosis.

**Fig. 3:**
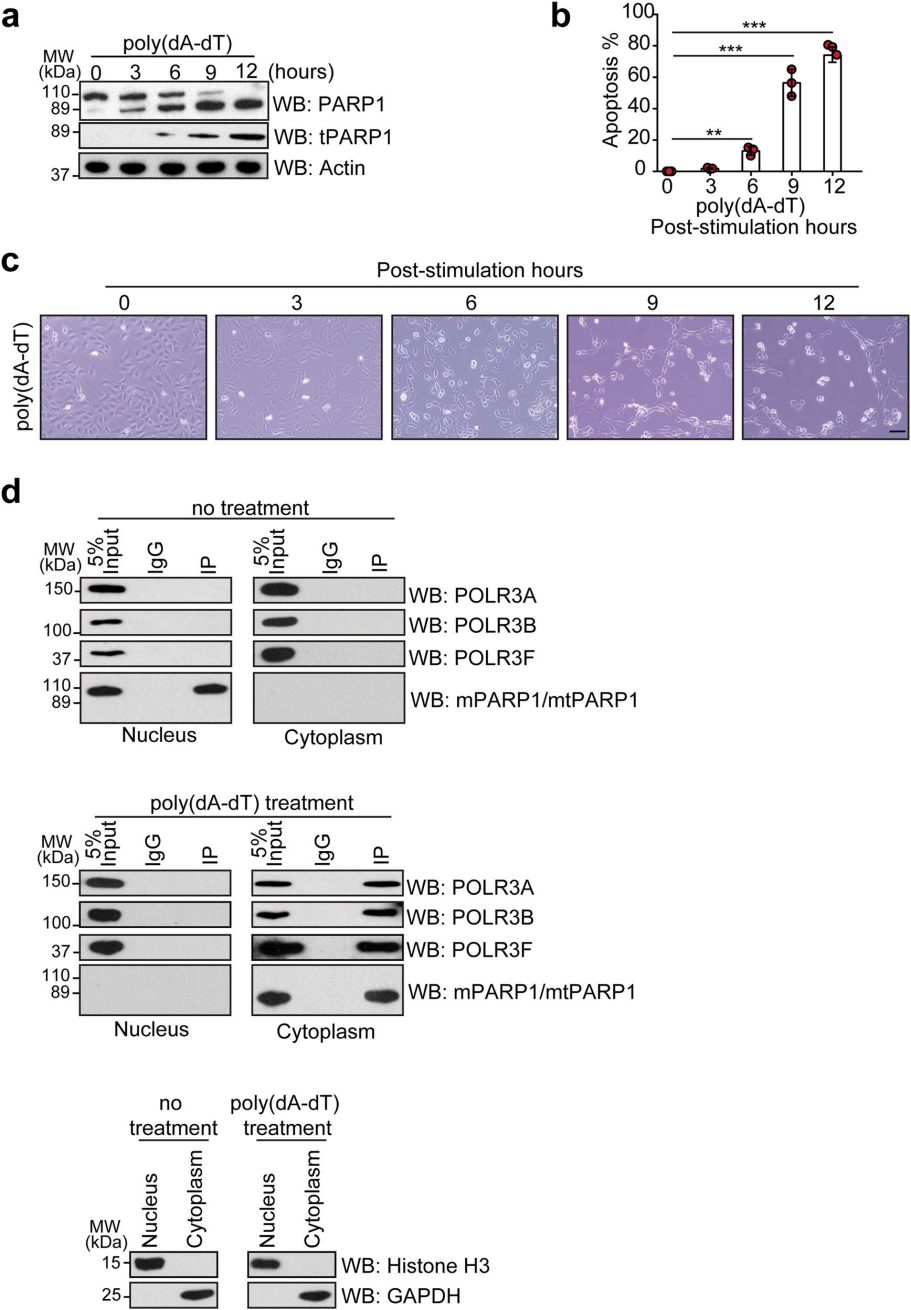
tPARP1 recognizes the Pol III complex during apoptosis. **a** The cleavage of PARP1 was examined in a time course in poly(dA-dT)-stimulated (3 μg/mL) apoptotic U2OS cells with anti-PARP1 N-terminus (PARP1) or C-terminus (tPARP1) antibody. **b** Apoptosis induced by 5 μg/mL of poly(dA-dT) in U2OS cells was determined by Annexin V-FITC/PI double staining. **c** Morphology change of apoptotic U2OS cells stimulated by 5 μg/mL poly(dA-dT) was recorded every 3 h for a total of 12 h. Scale bar, 50 μm. **d** mPARP1 was expressed in U2OS PARP1-null cells, followed by 5 μg/mL poly(dA-dT) induction for 6 h. mtPARP1 but not mPARP1 interacted with endogenous POLR3A, POLR3B, or POLR3F. Data are represented as means ± SD as indicated from three independent experiments. Significance of differences was evaluated by Student’s *t*-test. **P < 0.01; ***P < 0.001.

Since exogenous poly(dA-dT) mimics pathogenic DNA and has been used for examining the role of the Pol III complex in cytoplasm during apoptosis^51–53^, herein we transfected poly(dA-dT) into cells to validate apoptosis with three different approaches: (1) cleavage of PARP1 by caspase 3 was detected using two different antibodies, one antibody recognizing either full-length PARP1 or tPARP1, and the other antibody specifically detecting tPARP1 (Fig. 3a); (2) apoptotic cells were measured by Annexin V-fluorescein isothiocyanate (FITC) and propidium iodide (PI) in the fluorescence-activated cell sorting assay and the apoptotic cells were remarkably increased in response to poly(dA-dT) transfection (Fig. 3b and Supplementary Fig. S1); (3) the morphology changes were used to distinguish apoptotic cells when cells were transfected with poly(dA-dT) (Fig. 3c). Next, following caspase-mediated PARP1 cleavage, we found that mtPARP1 interacted with the Pol III subunits. However, without poly(dA-dT)-induced apoptosis, mPARP1 could not be converted into mtPARP1 and thus did not interact with the Pol III subunits (Fig. 3d). Collectively, tPARP1 recognizes the Pol III complex in cytoplasm during poly(dA-dT)-induced apoptosis.

### tPARP1 catalyzes ADP-ribosylation

**Fig. 4:**
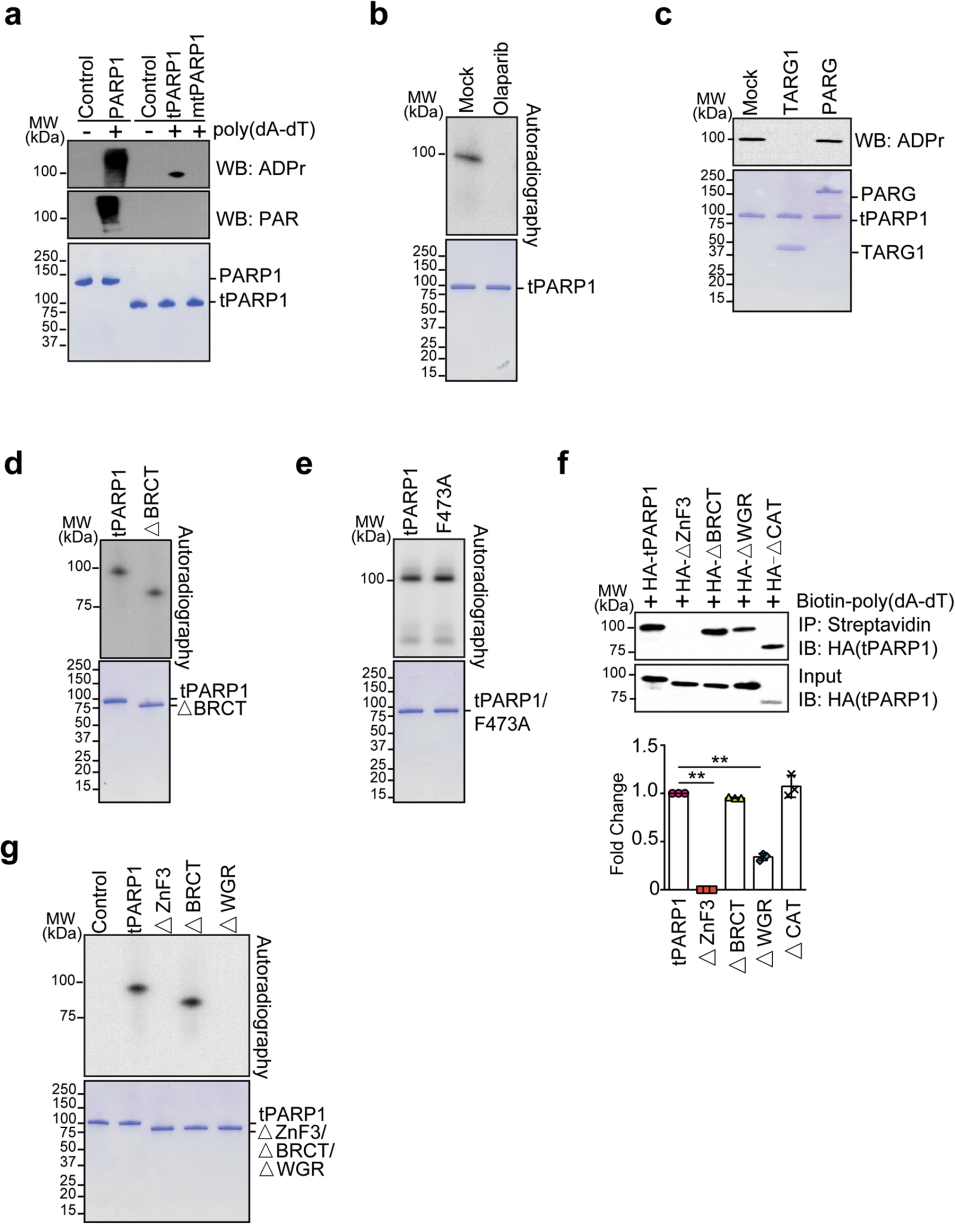
tPARP1 catalyzes ADP-ribosylation. **a** In vitro auto-ADP-ribosylation of PARP1 and tPARP1. Recombinant PARP1 or tPARP1 was incubated with NAD^+^ and poly(dA-dT). Auto-ADP-ribosylation was detected by anti-pan ADPr (upper panel) or anti-PAR (middle panel). Recombinant protein in each reaction was also examined by SDS-PAGE and Coomassie blue staining (lower panel). **b** Olaparib suppresses auto-ADP-ribosylation of tPARP1. **c** TARG1 but not PARG removes the auto-ADP-ribosylation of tPARP1. **d**, **e** The BRCT domain is not required for the auto-ADP-ribosylation of tPARP1. **f** HA-tagged full length of tPARP1 and four truncation mutants of tPARP1 were expressed in U2OS-PARP1 knockout cells, respectively. The cell lysates were extracted, followed by incubation with 5’ biotin-labeled poly(dA-dT). A streptavidin pull-down assay was performed and the complex was detected by western blotting (upper panel). Quantitative analysis of the levels of the pulled down proteins is shown in the lower panel. **g** The ZnF3 and the WGR domains are required for the auto-ADP-ribosylation of tPARP1. Data are represented as means ± SD as indicated from three independent experiments. Significance of differences was evaluated by Student’s *t-*test. **P < 0.01.

Since tPARP1 retains the catalytic domain, we examined whether tPARP1 had any enzymatic activity with auto-modification assays. The ADP-ribosylated tPARP1 was examined by sodium dodecyl sulfate-polyacrylamide gel electrophoresis (SDS-PAGE) followed by western blotting using anti-PAR or anti-ADPr antibody. As the positive control, the full-length PARP1 was activated by poly(dA-dT). The auto-PARylation was detected on the full-length PARP1. In contrast, we did not find auto-PARylated tPARP1. Instead, auto-ADP-ribosylated tPARP1 was detected by anti-ADPr antibody, which was also dependent on poly(dA-dT). Moreover, the E988A mutation abolished the auto-ADP-ribosylation of tPARP1, suggesting that tPARP1 is still able to catalyze ADP-ribosylation (Fig. 4a). To further validate the ADP-ribosylation on tPARP1, we treated the tPARP1 with olaparib, a potent PARP1 inhibitor, which competes NAD^+^ to occupy the catalytic pocket^54,55^. With olaparib treatment, we did not detect the auto-ADP-ribosylation of tPARP1 (Fig. 4b). Moreover, TARG1 but not PARG treatment was able to remove the auto-ADP-ribosylation of tPARP1, suggesting that auto-ADP-ribosylation is likely to be mono(ADP-ribosyl)ation (also known as MARylation) (Fig. 4c).

Next, we searched for the domains that are required for the auto-ADP-ribosylation. Of note, when we either deleted or mutated the BRCT domain from tPARP1 (tPARP1-ΔBRCT or BRCT F473A), we could still detect the auto-ADP-ribosylation, suggesting that the BRCT domain is not required for the enzymatic activity (Fig. 4d, e). To search for the regions in the tPARP1 that recognize poly(dA-dT), we incubated biotinylated poly(dA-dT) with cell lysates containing tPARP1 with each domain deletion and found that the ZnF3 domain was essential for the interaction with tPARP1. Moreover, deletion of the WGR domain also modestly impaired the binding of tPARP1 with poly(dA-dT), suggesting that the ZnF3 domain and the WGR domain may act together to recognize poly(dA-dT) (Fig. 4f). Consistently, lacking either the ZnF3 or the WGR domain suppressed the auto-ADP-ribosylation of tPARP1 (Fig. 4g).

### RNA Pol III subunits are substrates of tPARP1

**Fig. 5:**
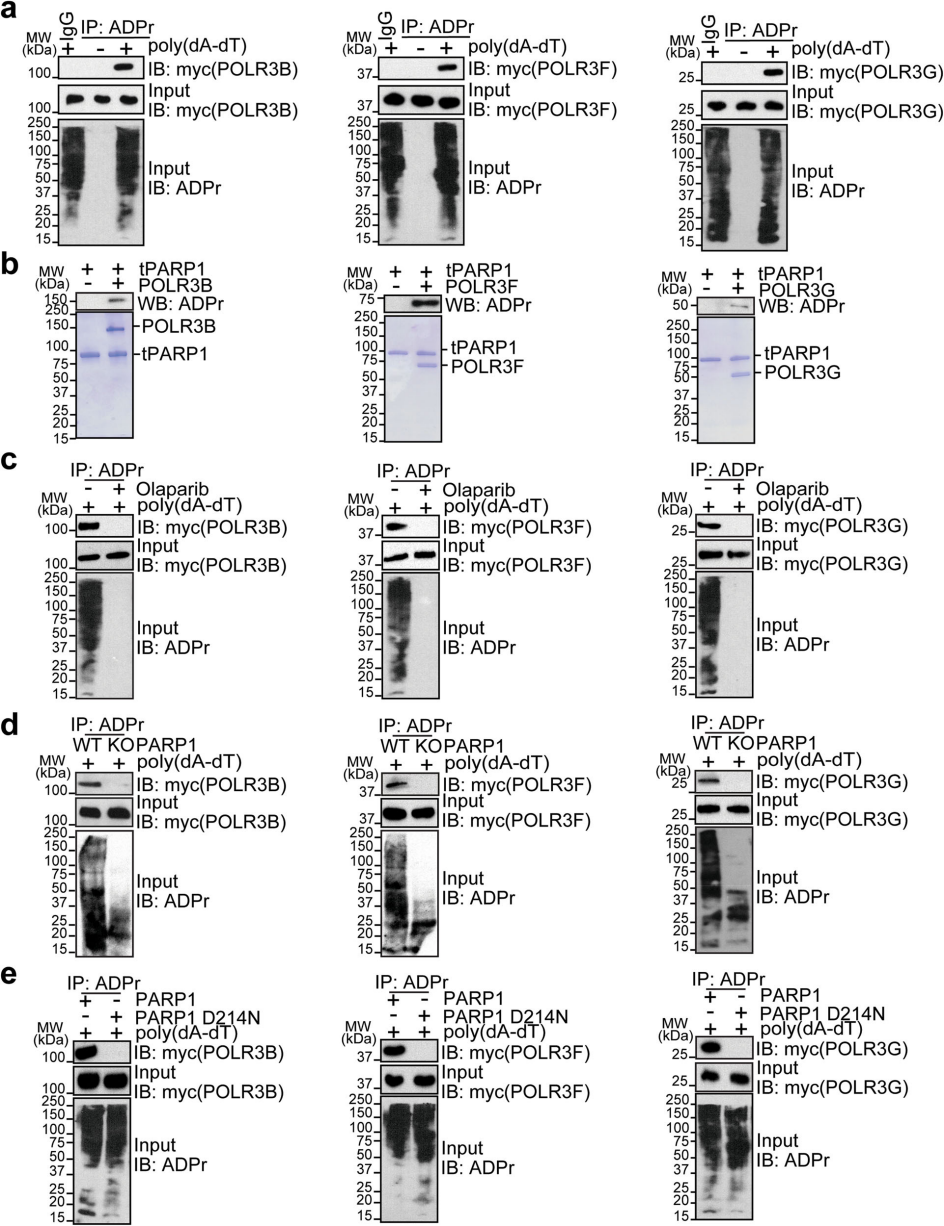
RNA Pol III subunits are substrates of tPARP1. **a** POLR3B, POLR3F, and POLR3G are identified as substrates of tPARP1. Individual subunits of Pol III with myc tag was expressed in U2OS cells, followed by poly(dA-dT)-stimulated apoptosis. Anti-ADPr antibody was used for IP; and the complex was analyzed by western blotting with anti-myc antibody. **b** POLR3B, POLR3F, and POLR3G are substrates of tPARP1 in vitro. Auto-ADP-ribosylation was detected by anti-ADPr antibody (upper panel). Recombinant protein in each reaction was also examined by the SDS-PAGE with Coomassie blue staining (lower panel). **c** ADP-ribosylation of POLR3B, POLR3F, or POLR3G was examined in U2OS cells treated with or without 1 µM olaparib for 1 h. **d** ADP-ribosylation of POLR3B, POLR3F, or POLR3G was examined in wild-type and PARP1 knockout U2OS cells. **e** ADP-ribosylation of POLR3B, POLR3F, or POLR3G was examined in U2OS cells expressing wild-type or caspase 3-resistant (D214N) PARP1. Note that the cells were harvested after 6-h treatment with 5 μg/mL poly(dA-dT) in **a**, **c**–**e**.

Since both mtPARP1 and mTARG1 can trap the Pol III complex for relatively stable interactions, we asked whether the Pol III complex was able to be ADP-ribosylated by tPARP1. We individually expressed nine subunits of Pol III including POLR3A, POLR3B, POLR3C, POLR3D, POLR3E, POLR3F, POLR3G, POLR3H, and POLR3K in U2OS cells, followed by poly(dA-dT) stimulation to induce apoptosis. ADP-ribosylated proteins were pulled down using anti-ADPr antibodies. We found ADP-ribosylation on POLR3B, POLR3F, and POLR3G by western blotting (Fig. 5a and Supplementary Fig. S2a, b). We also generated recombinant proteins of POLR3B, POLR3F, and POLR3G for in vitro ADP-ribosylation assays. The results showed that these three Pol III subunits can be ADP-ribosylated by tPARP1 (Fig. 5b).

Since olaparib suppresses the enzymatic activity of tPARP1, we treated the cells with olaparib and found that ADP-ribosylation of the Pol III subunits was suppressed (Fig. 5c). Moreover, the ADP-ribosylation of the Pol III subunits was also suppressed in cells lacking PARP1 (Fig. 5d). Since the cleavage of PARP1 occurs at D214, we mutated D214 into Asn (D214N) to inhibit caspase 3-mediated PARP1 cleavage (Supplementary Fig. S3a, b). In the cells only expressing the D214N mutant, the Pol III subunits could not be ADP-ribosylated during poly(dA-dT)-mediated apoptosis (Fig. 5e). Taken together, these results suggest that tPARP1 mediates ADP-ribosylation of the Pol III subunits during dsDNA-stimulated apoptosis.

Moreover, previous studies have showed that mouse PARP2 can be cleaved by caspase 3 at site ^58^-DNRD-^61^ ^56^, which raises the possibility that PARP2 may also be required for ADP-ribosylation of Pol III. However, suppression of ADP-ribosylation on Pol III only occurred in cells lacking PARP1 but not PARP2, suggesting that ADP-ribosylation on Pol III is mediated by tPARP1 (Supplementary Fig. S4a–c). In addition, we lysed the cells with 1% SDS to abolish any noncovalent ADP-ribosylation on Pol III but still found ADP-ribosylation on Pol III, suggesting that ADPr is covalently associated with Pol III (Supplementary Fig. S5a–c). Moreover, we treated ADP-ribosylated Pol III with NH_2_OH to remove the covalently linked ADPr and found that ADP-ribosylation was completely removed, further confirming that ADPr is covalently linked with Pol III (Supplementary Fig. S6a–c).

To further validate the ADP-ribosylation on Pol III is MARylation but not PARylation, we treated ADP-ribosylated proteins with TARG1 or PARG and found that only TARG1 but not PARG was able to remove ADP-ribosylation on Pol III (Supplementary Fig. S7a–c), indicating that this ADP-ribosylation event is likely to be multiple mono-ADP-ribosylation events or oligo-ADP-ribosylation events.

### tPARP1 enhances the activity of cytosolic Pol III during apoptosis

**Fig. 6:**
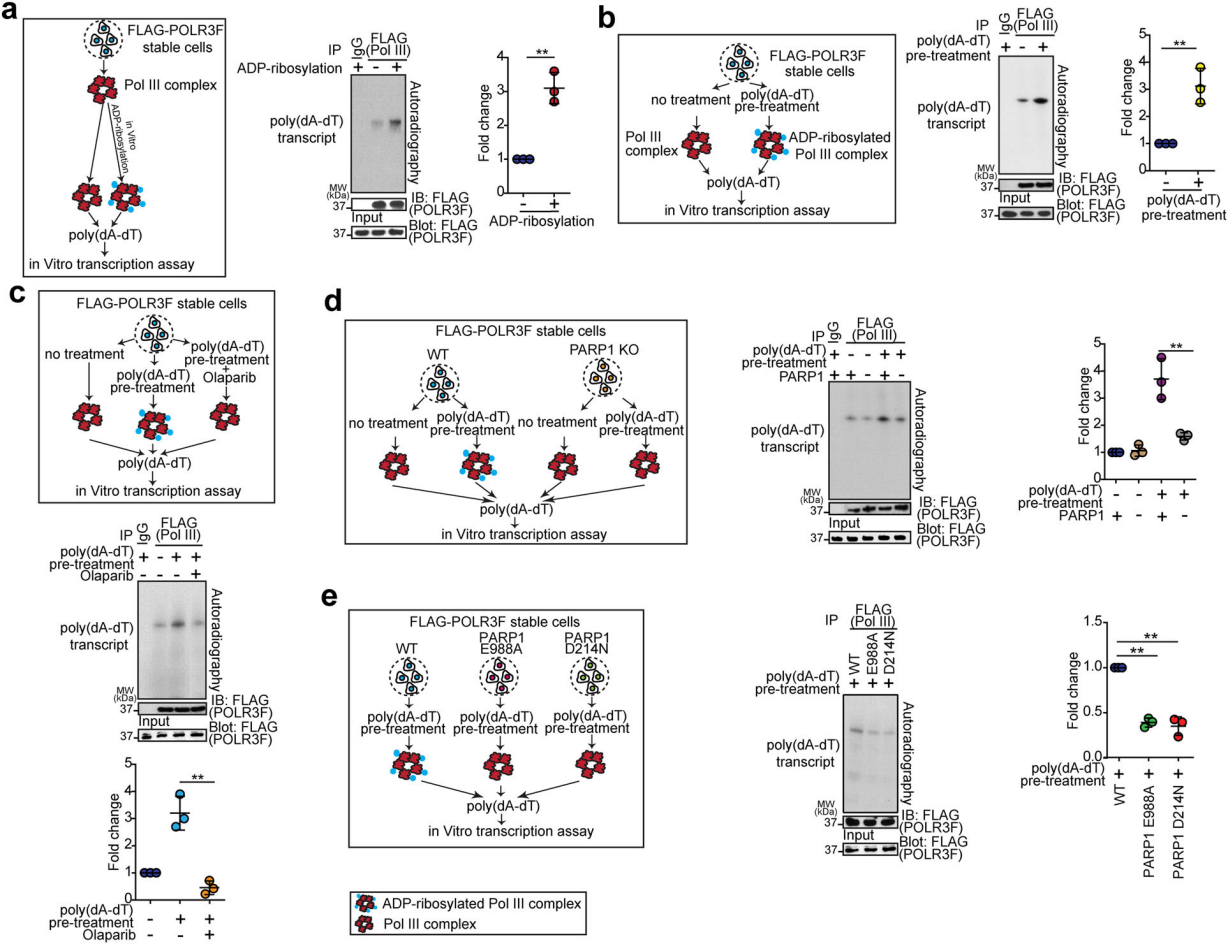
tPARP1 enhances the activity of Pol III during apoptosis. **a** The enzymatic activity of ADP-ribosylated Pol III was examined through in vitro Pol III transcription assay. The Pol III complex was purified from U2OS cells stably expressing FLAG-POLR3F and ADP-ribosylated by tPARP1 in vitro. In vitro Pol III transcription assay was performed with ^32^P-UTP. The expression of POLR3F was used as a loading control for IP. IgG was used as the negative control for IP in lane one. **b** The enzymatic activity of Pol III in apoptotic cells was examined. U2OS cells stably expressing FLAG-POLR3F were treated with or without poly(dA-dT). **c** The enzymatic activity of Pol III in apoptotic cells was examined when U2OS-POLR3F cells were treated with or without 1 µM olaparib for 1 h. **d** The enzymatic activity of Pol III was examined in wild-type or PARP1 knockout U2OS cells. **e** The enzymatic activity of Pol III was examined in the PARP1-null cells reconstructed with wild-type PARP1, the E988A mutant (catalytic-dead mutant), or the D214N mutant (caspase 3-resistant mutant). Note that the apoptotic cells were harvested after 6-h treatment with 5 μg/mL poly(dA-dT) in **b**–**e**. Data are represented as means ± SD as indicated from three independent experiments. Significance of differences was evaluated by Student’s *t*-test. **P < 0.01.

Next, we examined the function of tPARP1-mediated ADP-ribosylation on Pol III. It has been shown that Pol III senses foreign dsDNA in the cytosol and transcribes 5’ triphosphate (5’ppp) RNA^49,50^. To examine the enzymatic activity of the Pol III complex, we purified Pol III from cell lysates, which was able to use poly(dA-dT) as the template to transcribe dsRNA. We then ADP-ribosylated the purified Pol III with tPARP1. Interestingly, the enzymatic activity of ADP-ribosylated Pol III was significantly increased during dsRNA transcription (Fig. 6a). Moreover, we purified the Pol III complex from cells undergoing poly(dA-dT)-induced apoptosis. Compared to the mock-treated cells, the Pol III complex from apoptotic cells had enhanced enzymatic activity for dsRNA transcription (Fig. 6b). When we treated the cells with olaparib to suppress the activity of endogenous tPARP1, the enzymatic activity of Pol III complex isolated from apoptotic cells was also reduced (Fig. 6c). In addition, in the cells lacking PARP1, the enzymatic activity of Pol III complex was lower than that in the cells re-expressing the full-length PARP1 during poly(dA-dT)-induced apoptosis (Fig. 6d), and neither the E988A nor the D214N mutant could rescue the loss of transcription activity of the Pol III complex (Fig. 6e). Taken together, these results suggest that tPARP1-mediated ADP-ribosylation facilitates the enzymatic activity of Pol III complex towards dsRNA transcription during apoptosis.

In addition, Pol III is known to regulate other small RNA transcription. Thus, we also examined the potential impact of ADP-ribosylation on other Pol III-dependent RNA transcription. We randomly selected several tRNAs, U6 snRNA, and 5S rRNA and examined their expression in U2OS cells treated with poly(dA-dT). However, we did not observe obvious changes in the transcription of these conventional Pol III targets (Supplementary Fig. S8 and Table S3). Since transcription of these targets is mediated by nuclear Pol III, ADP-ribosylation of cytoplasmic Pol III does not affect these transcription events.

### ADP-ribosylation of Pol III facilitates its enzymatic activity

Our studies suggest that Pol III is a substrate of tPARP1. From the published database, E100 of POLR3F is known to be ADP-ribosylated^57^ (Supplementary Fig. S9a). Thus, we explored whether ADP-ribosylated E100 of Pol III regulates the activity of Pol III. We mutated E100 into Ala and generated POLR3F E100A-expressing cell line. First, we found that E100A mutation was unable to be ADP-ribosylated by tPARP1 in vitro (Supplementary Fig. S9b). Next, to exclude the possible structural and functional defect in the E100A mutant, in vitro transcription assay was performed with E100A. The results showed that the enzymatic activity of E100A mutant on poly(dA-dT) was similar with the wild type, indicating that the enzymatic activity of E100A mutation on transcription without poly(dA-dT) pretreatment was not affected (Supplementary Fig. S9c). In addition, the general E100A mutant Pol III activities on tRNAs, U6 snRNA, and 5S rRNA were also tested. Again, we did not observe obvious changes in the transcription of these conventional Pol III targets (Supplementary Fig. S9d).

Next, we explored the potentially direct link between ADP-ribosylation on POLR3F and expression of IFN-β induction and found that the E100A mutation impaired the activity of Pol III and the IFN-β induction when cells were stimulated by poly(dA-dT) (Supplementary Fig. S10a, b). To further understand the molecular mechanism of this ADP-ribosylation event, we modeled the structure of human Pol III complex based on the known structure of yeast Pol III complex^58–61^. Compared to the yeast Pol III complex, human Pol III complex is highly conserved with an almost identical active center for RNA synthesis. Both yeast and human Pol III complexes comprise 17 subunits, and 9 subunits of them form a structure core. The largest subunits POLR3A and POLR3B comprise the active center of Pol III. POLR3F is a core component of transcription initiation complex (PDB: 6CNB) (Supplementary Fig. S10c). Moreover, POLR3E–POLR3F and TFIIIB structurally form a sub-complex (Supplementary Fig. S10d). The residue E100 is located in a pocket of POLR3F (dashed circle in Supplementary Fig. S10e) that may impact the complex conformation on POLR3E and TFIIIB. Once the ADP-ribosylation occurs on E100 of POLR3F (Supplementary Fig. S10f), it might enlarge the pocket and subsequently leads to a more open conformation of the POLR3E–POLR3F–TFIIIB complex. These structural rearrangements induced by ADP-ribosylation on E100 may sequentially impact the catalytic center by enclosing the catalytic residues to the template DNA through the pack of neighboring protein subunits, including POLR3A, POLR3B, and POLR3K (Supplementary Fig. S10c). Thus, this ADP-ribosylation event likely increases processivity and promotes the activity of Pol III, functioning as a conformational augmenter during Pol III-mediated poly(dA-dT) transcription initiation and elongation.

Moreover, we examined whether ADP-ribosylation abolishes any subunit in the Pol III complex. We treated Pol III complex with ADP-ribosylation in vitro. However, we did not observe any dissociation of the complex (Supplementary Fig. S11a). In addition, we stimulated cells with poly(dA-dT) to induce ADP-ribosylation and purified cytosolic Pol III complex from unstimulated cells and stimulated cells. Again, we did not detect obvious difference of each Pol III subunit between these two treatments (Supplementary Fig. S11b). Collectively, the ADP-ribosylation on Pol III does not induce dissociation of the Pol III complex.

### tPARP1 contributes to the IFN response and promotes apoptosis

**Fig. 7:**
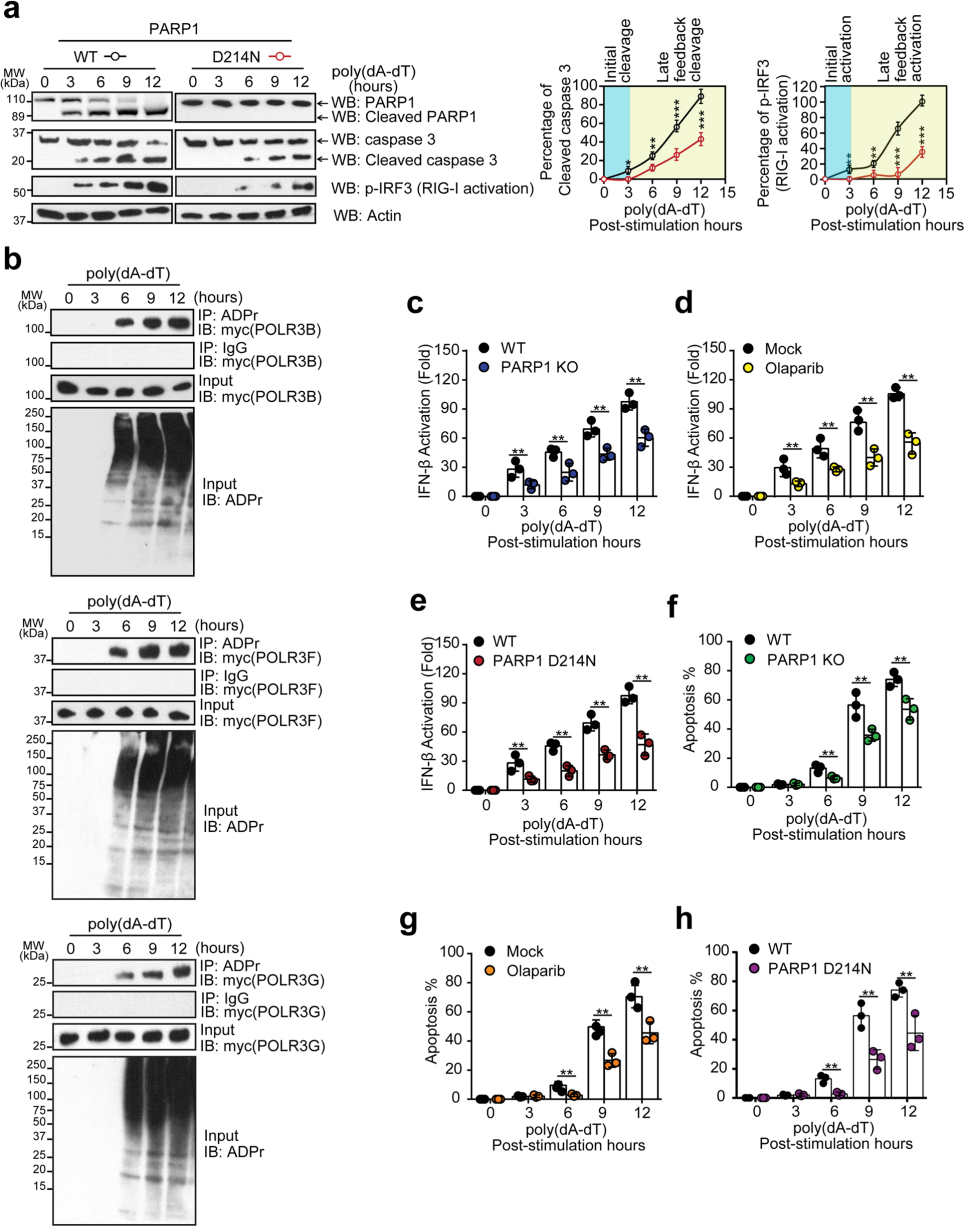
PARP1 cleavage promotes apoptosis via a positive feedback loop. **a** PARP1 cleavage, caspase 3 activation, and RIG-I activation were examined in wild-type U2OS cells and U2OS cells expressing non-cleavable PARP1 (D214N) at different time points during 5 μg/mL poly(dA-dT)-induced apoptosis. **b** Myc-tagged POLR3B, POLR3F, or POLR3G subunit was expressed in U2OS cells. ADP-ribosylation of Pol III was examined by IP and western blotting with the indicated antibodies in a time course in cells treated with 5 μg/mL poly(dA-dT). **c** Induction of IFN-β by poly(dA-dT) was analyzed by luciferase reporter assay in wild-type or PARP1-null cells. **d** Induction of IFN-β by poly(dA-dT) was examined in U2OS cells with or without 1-h treatment with 1 µM olaparib. **e** Induction of IFN-β by poly(dA-dT) was determined in PARP1-null cells reconstructed with wild-type PARP1 or the D214N mutant. **f** Apoptotic cells were examined by Annexin V-FITC/PI double staining in parental U2OS cells or PARP1 knockout cells. **g** Apoptotic cells were examined by Annexin V-FITC/PI double staining in U2OS cells with or without 1-h treatment with 1 µM olaparib. **h** Apoptotic cells were examined by Annexin V-FITC/PI double staining in the PARP1-null cells reconstructed with wild-type PARP1 or the D214N mutant. Note that the apoptotic cells were harvested after 6-h treatment with 5 μg/mL poly(dA-dT) in **c**–**h**. Data are represented as means ± SD as indicated from three independent experiments. Significance of differences was evaluated by Student’s *t*-test. *P < 0.05; **P < 0.01; ***P < 0.001.

Caspase 3-mediated PARP1 cleavage is a hallmark for the middle stage of apoptosis^3^. Since we knew that tPARP1 mediates ADP-ribosylation on Pol III, we wondered whether this ADP-ribosylation event regulates apoptosis. Interestingly, Pol III is a foreign dsDNA sensor in the cytoplasm. Once Pol III is activated, it induces innate immune response such as stimulating the expression of IFN-β via RIG-I-dependent pathway^50,52^. It facilitates apoptosis of host cells, which prevents pathogen propagation^49,50^. Thus, it is possible that tPARP1-mediated Pol III ADP-ribosylation forms a positive feedback loop to facilitate apoptosis. To examine the role of tPARP1-mediated ADP-ribosylation in this biological process, we carefully examined the tPARP1 generation, the activation of caspase 3, ADP-ribosylation of Pol III, and activation of the RIG-I pathway. We found that, 3-h following poly(dA-dT) treatment, PARP1 started to be cleaved by caspase 3 (Fig. 7a). Meanwhile, cleavage of pro-caspase 3 was observed, which is a surrogate marker of the activation of pro-caspase 3 (Fig. 7a), suggesting that caspase-mediated protein cleavage occurs in a relatively early phase of apoptosis. These events were associated with the IRF3 phosphorylation (Fig. 7a), a surrogate marker of the activation of the RIG-I pathway. Because of the generation of tPARP1, ADP-ribosylation of Pol III was found 6 h following poly(dA-dT) treatment (Fig. 7b). Twelve hours following poly(dA-dT) treatment, PARP1 cleavage, and activation of caspase 3, both ADP-ribosylation of Pol III and activation of the RIG-I pathway were progressively increased (Fig. 7a, b). However, in the cells that only expressed the non-cleavable PARP1 (the D214N mutant), the activation of caspase 3 and RIG-I were significantly delayed (Fig. 7a). Deficiency of PARP1 also suppressed the RIG-I activation in the apoptotic cells (Supplementary Fig. S12). Moreover, direct RIG-I stimulation (5’ triphosphate double-stranded RNA) was also compared with poly(dA-dT) under the same condition through examining the PARP1 cleavage, RIG-I activation, and IFN-β induction, respectively. As a result, we did not observe any significant difference in the two treatments (Supplementary Fig. S13a, b).

Moreover, we used an established luciferase reporter system to measure the level of IFN-β^62^. When cells were transfected with poly(dA-dT) to induce apoptosis, the level of IFN-β was reduced in PARP1-deficient cells compared with that in the wild-type cells (Fig. 7c). Moreover, when cells were pretreated with olaparib to suppress the enzymatic activity of PARP1, the level of IFN-β was decreased as well (Fig. 7d). To further confirm the role of tPARP1 in this process, we compared cleavable and non-cleavable (D214N) forms of the full-length PARP1. We found that only the D214N mutant but not the wild-type caspase-cleavable PARP1 suppressed the generation of IFN-β in poly(dA-dT)-mediated apoptotic cells (Fig. 7e). The cells were also treated with 30 μM Pol III inhibitor ML-60218 for 2 h to inhibit the transcription activity of Pol III, and then the IFN-β production was examined. We found that the level of IFN-β was barely detected (Supplementary Fig. S14). Collectively, these results suggest that the enzymatic activity of tPARP1 facilitates the generation of IFN-β in apoptotic cells.

To further confirm the role of tPARP1-mediated ADP-ribosylation in the production of IFN-β, real-time reverse transcription-quantitative PCR (RT-qPCR) was also conducted to examine the endogenous transcription of IFN-β. Consistent with the results from the luciferase reporter assays, we found that ADP-ribosylation indeed facilitated endogenous transcription of IFN-β during apoptosis (Supplementary Fig. S15a–c). It has been shown that PARP9 and PARP14 regulate IFN-β expression during tissue inflammation^63^. To test whether PARP9 or PARP14 plays a role in the IFN-β induction during apoptosis, PARP9-null cells and PARP14-null cells were generated. Comparing with wild-type cells, we did not observe obvious changes of IFN-β expression in PARP9-null cells or PARP14-null cells (Supplementary Fig. S16a–c).

The expression level of IFN-β plays an important role in the clearance of infected cells, to ensure the survival of organism. Here we further investigated whether PARP1 facilitates poly(dA-dT)-triggered apoptosis. Consistent with those observations of the role of tPARP1 in IFN-β production, PARP1 with its enzymatic activity promoted poly(dA-dT)-induced apoptosis, but not the caspase-resistant PARP1 (Fig. 7f–h and Supplementary Fig. S17).

To exclude the possibility of clonal variation phenomena in the engineered cells generated, a set of experiments were performed to investigate the difference between parental and engineered cells in apoptosis triggered by tumor necrosis factor (TNF), TRAIL, FasL, and H_2_O_2_, in addition to poly(dA-dT). The results showed no significant difference with apoptosis induction in all the tested engineered cells, compared with parental cells (Supplementary Fig. S18a, b).

The major pattern recognition receptors (PRRs) sensing cytosolic dsDNA are RIG-I, cGAS, MDA5, DAI, AIM2, and NALP3. However, both AIM2 and NALP3 specifically activate inflammasome to induce IL-1, not IFN-β. To investigate the activation of non-RIG-I PRRs in cells, in addition to the RIG-I pathway, we treated the cells with poly(dA-dT), and the activation of cGAS, MDA5 and DAI were detected by IRF3 phosphorylation using western blotting. The result showed that loss of cGAS, MDA5, or DAI drastically repressed IRF3 activation and sequentially impaired the induction of IFN-β. However, compared to the wide-type U2OS cells, no significant difference of cGAS-, MDA5-, and DAI-activated IRF3 and IFN-β production was observed in the FLAG-POLR3F U2OS cells, indicating that the engineered cell lines used in our study functioned well without any off-target defect (Supplementary Fig. S19a, b). Additionally, the role of RIG-I pathway in caspase activation, IRF3 phosphorylation, and IFN production was examined, and a significant reduction of caspase 3 cleavage, IRF3 phosphorylation, IFN-β production, and apoptosis in cells lacking RIG-I after treatment with poly(dA-dT) was observed (Supplementary Fig. S20a–c).

To recapitulate the pathogen invasion-induced apoptosis, we also infected Raw264.7 cells with adenovirus to induce apoptosis (Supplementary Fig. S21a, b). Adenovirus is a DNA virus that is recognized by Pol III and activates IFN-β expression by RIG-I-dependent pathway^50^. Again, IFN-β induction by adenovirus was markedly reduced in PARP1-deficient cells or non-cleavable PARP1 (D214N)-expressing cells (Supplementary Fig. S21c, d), suggesting that PARP1 cleavage promotes IFN-β expression and apoptosis during pathogen infection.

## Discussion

**Fig. 8:**
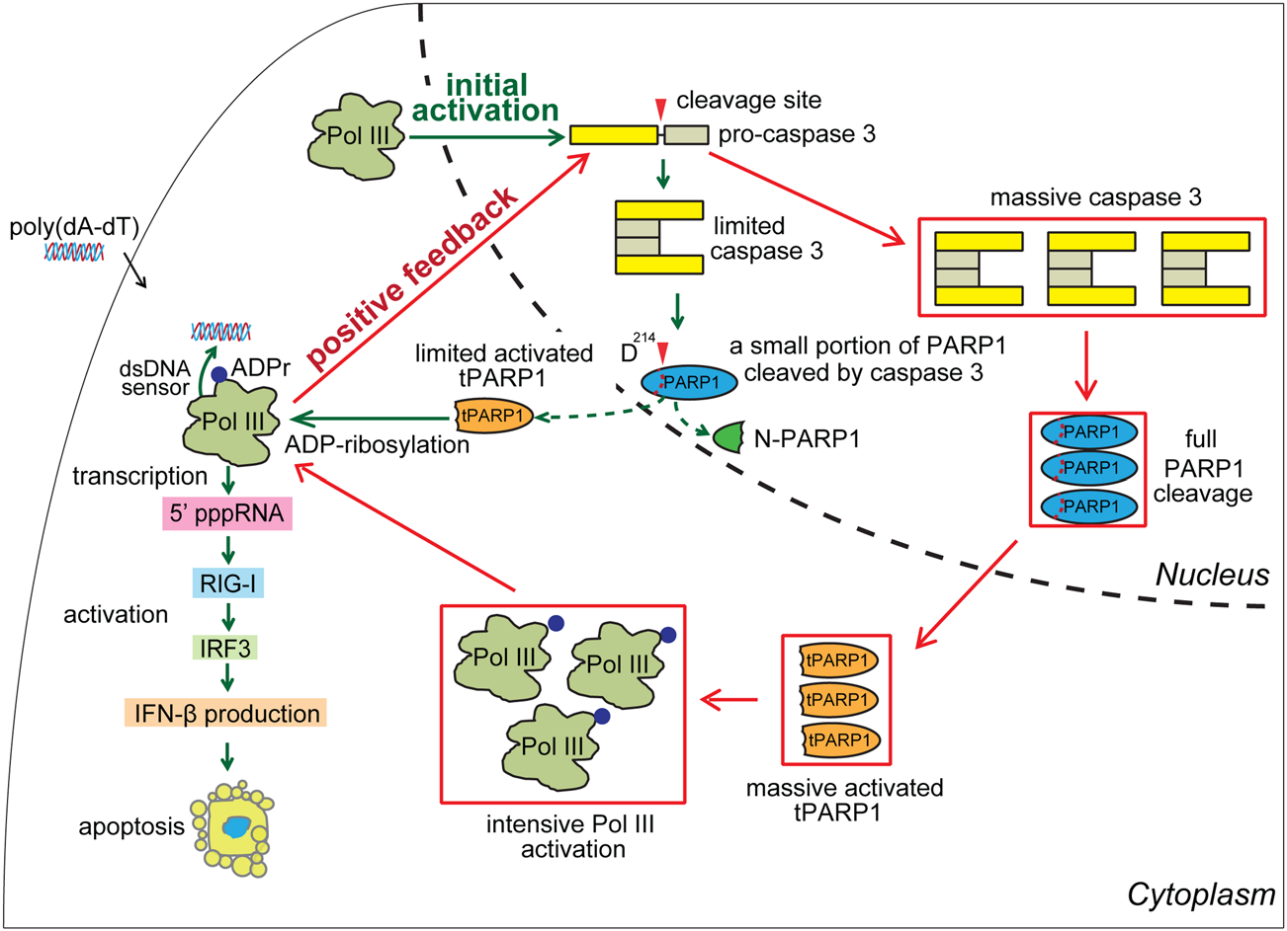
A model of tPARP1-mediated RNA Pol III ADP-ribosylation forms a positive feedback loop to facilitate apoptosis. Pol III mediates the activation of caspase 3 during the early stage of apoptosis. The early activation of caspase 3 digests a portion of PARP1. Cleaved PARP1 relocates from nucleus to cytoplasm to catalyze ADP-ribosylation on Pol III in cytoplasm, which in turn activates Pol III and RIG-I-dependent apoptotic pathways. ADP-ribosylated Pol III induces a positive feedback loop to further activate caspase 3 and PARP1 cleavage. Eventually, this positive feedback loop accelerates apoptosis. The green lines represent the initial activation of caspase 3, limited cleavage of PARP1, and early-phase activation of the RIG-I pathway. The red lines represent the positive feedback loop via ADP-ribosylation of Pol III, full activation of caspase 3, and the RIG-I pathway to accelerate apoptosis.

In this study, we have shown that tPARP1 is able to ADP-ribosylate Pol III, which facilitates the activation of Pol III in a positive feedback loop for innate immune response during apoptosis (Fig. 8). tPARP1 is generated by caspase 3-mediated cleavage during apoptosis^8,10,11,39^. It has been hypothesized that tPARP1 is a catalytic-dead protein^23^. Here we demonstrate that tPARP1 is still an enzyme that catalyzes at least MARylation. Compared to the full-length PARP1, although tPARP1 lacks the two N-terminal zinc finger motifs, it still retains rest of the domains including the ZnF3 domain, the BRCT domain, the WGR domain as well as the C-terminal catalytic domain^64^, which resembles PARP1 in lower eukaryotes^43–45^. The tPARP1-mediated ADP-ribosylation is activated by poly(dA-dT), which is recognized by the ZnF3 domain and the WGR domain of tPARP1. It is likely that the binding of tPARP1 with poly(dA-dT) induces the intra-molecular conformational changes resulting in its activation. Thus, we expect that, without the first two zinc finger motifs, the interaction site on the BRCT domain is exposed for the tPARP1-mediated ADP-ribosylation on Pol III. However, this activation is different from the PARP1-mediated PARylation^20,21,23,46,65^. It is probably because the activation mode in the catalytic domain of tPARP1 is slightly different from that in the full-length protein, which does not allow the formation of ADPr polymers^65^. It would be intriguing to see the detailed activation mechanism from structural analysis on tPARP1 in the future.

Nevertheless, tPARP1 and TARG1 have the same targets, namely, Pol III, in cells. Since TARG1 removes MARylation from protein substrates^46,48^, it is likely that tPARP1 indeed MARylates Pol III, which is confirmed by the results from our in vivo and in vitro assays. Interestingly, following caspase 3-mediated cleavage, tPARP1 translocates to the cytoplasm during apoptosis^37–42^ where Pol III relocates for sensing foreign DNA^49–51,66^. However, it has been shown that Pol III senses foreign DNA and mediates the activation of caspase 3 during the early stage of apoptosis^52^, which is an upstream event of PARP1 cleavage. Here, by carefully examining PARP1 cleavage, activation of Pol III, and its downstream RIG-1-dependent pathway, we speculate that tPARP1-induced Pol III ADP-ribosylation is one of the positive feedback loops promoting apoptosis. Without ADP-ribosylation, Pol III is still able to sense the dsDNA from invaded pathogens and activates downstream RIG-I-dependent apoptotic pathways. However, without ADP-ribosylation, activation of downstream caspase 3 could be very limited, which induces digestion of a small portion of PARP1. Since PARP1 is one of the most abundant proteins in the cell and is very potent to induce protein ADP-ribosylation, even a small portion of cleaved PARP1 is sufficient to catalyze ADP-ribosylation on Pol III in cytoplasm, which in turn further activates RIG-I-dependent apoptotic pathways. This positive feedback loop accelerates apoptosis (Fig. 8). Since Pol III activates innate immune response for accelerating apoptosis in the host cells^7,52^, these results demonstrate that tPARP1-mediated ADP-ribosylation facilitates apoptosis. Once the apoptosis starts and PARP1 is cleaved, it is an irreversible biological process. Thus, our study reveals a pivotal biological function of tPARP1 in apoptosis.

In our study, we also performed preliminary structural modeling analysis and found that the ADP-ribosylation on E100 of POLR3F may induce conformational changes in the whole complex and regulate the enzymatic activity of Pol III. Moreover, ADP-ribosylation brings negative charges to the protein substrates^14,16,19,27^. It is possible that ADP-ribosylation-associated charges also fine-tune the tertiary structure of the Pol III complex for the activation^58–61,67^. In addition to E100 of POLR3F, other sites on Pol III may also be ADP-ribosylated. We have shown that tPARP1 ADP-ribosylates several subunits of the Pol III complex. It would be interesting to reveal the detailed molecular mechanism by structure analysis in the future. Here we have only revealed the Pol III complex components as substrates of tPARP1. We cannot rule out other potential substrates of tPARP1 in addition to Pol III that may also facilitate apoptosis. Future proteomic analysis may reveal other substrates of tPARP1.

Collectively, our results demonstrate that tPARP1 is an enzyme that catalyzes ADP-ribosylation on the Pol III complex for the activation of innate immune response and promoting apoptosis.

## Materials and methods

### Cell lines, transfection, and protein reconstitutions

293T cells, U2OS cells, and Raw264.7 cells were purchased from ATCC and cultured in Dulbecco’s modified Eagle’s medium (DMEM) containing 10% fetal bovine serum at 37 °C with 5% CO_2_. Cells were confirmed negative for mycobacteria contamination using the MycoAlert Mycoplasma Detection Kit (Lonza).

Transfection of the cells was carried out using Lipofectamine 2000 (Thermo Fisher Scientific) according to the manufacturer’s protocol. Poly(dA-dT) was purchased from Sigma and re-suspended in the sterile phosphate-buffered saline (PBS) buffer. Poly(dA-dT) was incubated with the indicated amounts of Lipofectamine 2000. The mixture was applied into the cells. 5’ppp dsRNA was purchased from InvivoGen.

For apoptosis measurement, cells were incubated with TNF (20 nM, 3 h), TRAIL (200 ng/mL, 24 h), soluble recombinant human FasL (1 μg/mL, 12 h), and H_2_O_2_ (200 μM, 24 h) under standard cell culture conditions, respectively.

To establish cells stably expressing mtPARP1, mPARP1, mPARG, or mTARG1, plasmids of SFB-tPARP1 E988A, SFB-PARP1 E988A, SFB-PARG E756A, or SFB-TARG1 D125A were transfected into 293T cells, followed by 1 µg/mL puromycin selection for 2 weeks. To establish cells stably expressing enzymatic-dead enzymatic PARP1 or caspase 3 cutting-resistant PARP1, PARP1-deficient U2OS cells were reconstituted with HA-PARP1 E988A or HA-PARP1 D214N constructed in pcDNA3.1/Hygro(+) vector, followed by 300 µg/mL hygromycin B selection. U2OS cells stably expressing FLAG-POLR3F were selected with 400 µg/mL of G418 for 2 weeks. PARP9 small interfering RNA (siRNA; Catalog #AM16708) was purchased from Thermo Fisher Scientific. Western blot analysis was conducted to validate the efficiency of the reconstitution of the indicated protein. To generate knockout cell lines, cells were transfected with PX459 vector containing cGAS-, MDA5-, or DAI-sgRNA for target protein knockout. Transfected cells were plated at low density in 1.5 μg/mL puromycin. Single colonies were propagated, and individual clones were analyzed by western blotting.

### Vector constructs and antibodies

To generate myc-, HA-, and GST-tagged proteins, DNA fragment containing *POLR3A*, *POLR3B*, *POLR3C*, *POLR3D*, *POLR3E*, *POLR3F*, *POLR3G*, *POLR3H*, and *POLR3K* were cloned in-frame with tags into pcDNA3.1-myc, pCMV-HA, or pGEX-4T-1 vectors, respectively. POLR3A-, POLR3B-, or PARG-coding sequence was cloned into pFastBac vector with GST tag to generate recombinant proteins. TARG1 was also cloned into pGEX-4T-1 vector. mtPARP1, mutant PARP1, mutant PARG, and mutant TARG1 were cloned into pIRES2-SFB vector. Deletion mutants or point mutations were introduced by Quikchange site-directed mutagenesis and confirmed by Sanger sequencing. pSpCas9(BB)-2A-Puro (PX459) V2.0 (plasmid #62988) and pCW-Cas9 (plasmid #50661) were purchased from Addgene. The sgRNA sequence for cGAS knockout was 5′-CGGCCCCCATTCTCGTACGG-3′. The sgRNA sequence for MDA5 knockout was 5′-AACTGCCTGCATGTTCCCGG-3′. The sgRNA sequence for DAI knockout was 5′-GGACGATTTACCGCCCAGGT-3′.

Anti-β-actin monoclonal antibody (Cat #A2228) and anti-FLAG monoclonal antibody (Cat #F1804) were purchased from Sigma. Anti-HA monoclonal antibody (Cat #MMS-101R) was from Covance. Anti-HA polyclonal antibody (Cat #ab9110) was from Abcam. Anti-myc antibody (9E10) (Cat #13-2500) was from Thermo Fisher Scientific. Anti-poly(ADPr) monoclonal antibody (Cat #4335-MC-100) was purchased from Trevigen. Anti-ADPr antibody was generated in-house. Both anti-PARP1 antibody (Cat #ab227244) and anti-cleaved PARP1 antibody (Y34) (Cat #ab32561) were from Abcam. Annexin V-FITC (Cat #556419) was from BD Biosciences. Anti-POLR3A polyclonal antibody (Cat #PA5-58170), anti-POLR3B polyclonal antibody (Cat #PA5-99691), anti-POLR3C monoclonal antibody (OTI2H1) (Cat #MA5-26051), anti-POLR3D polyclonal antibody (Cat #PA5-64342), anti-POLR3E polyclonal antibody (Cat #PA5-59585), anti-POLR3F polyclonal antibody (Cat #PA5-20589), anti-POLR3G polyclonal antibody (Cat #24701-1-AP), anti-POLR3H polyclonal antibody (Cat #PA5-61325), and anti-POLR3K polyclonal antibody (Cat #PA5-103798) were from Thermo Fisher Scientific. Anti-Histone H3 polyclonal antibody (Cat #06-755) was purchased from Millipore Sigma. Anti-GAPDH monoclonal antibody (Cat #MA5-15738) and anti-caspase 3 monoclonal antibody (3CSP01 (7.1.44)) (Cat #MA5-11516) were from Thermo Fisher Scientific. Both anti-IRF3 monoclonal antibody (EPR2418Y) (Cat #ab68481) and anti-IRF3 (phospho S386) monoclonal antibody (EPR2346) (Cat #ab76493) were from Abcam. Anti-PARP9 polyclonal antibody (Cat #17535-1-AP) was from Thermo Fisher Scientific. Anti-PARP14 monoclonal antibody (C-1) (Cat #sc-377150) was from Santa Cruz Biotechnology.

### Recombinant protein production

All recombinant proteins were expressed in BL21 cells, except POLR3A and POLR3B. Baculoviruses expressing GST-POLR3A and GST-POLR3B were prepared using Bac-to-Bac system according to the manufacturer’s protocols. Proteins were expressed in SF9 insect cells by infection with the Baculoviruses for 2 days. GST fusion proteins were purified using Glutathione Sepharose 4B. His-tagged tPARP1 or full-length PARP1 was purified using Ni^2+^-NTA chromatography. All recombinant proteins were examined by SDS-PAGE followed by Coomassie blue staining.

### In vitro ADP-ribosylation assay

The auto-ADP-ribosylation assay was performed using 50 nM PARP1 and tPARP1 with 500 nM poly(dA-dT) in PAR reaction buffer (100 mM Tris-HCl, pH 8.0, 150 mM NaCl, 10 mM MgCl_2_, 500 µM dithiothreitol (DTT), and 0.125 μM ^32^P-NAD^+^ or 0.25 μM NAD^+^). The reaction was carried out for 20 min at 30 °C and stopped by the addition of SDS-loading buffer. The products were separated in SDS-PAGE gel followed by western blotting using anti-PAR or anti-pan ADPr antibody or subjected to autoradiography. The protein in each reaction was stained by Coomassie blue. Recombinant PARG or TARG1 was added to the mixture for another 30 min to remove ADP-ribosylation, and the products were analyzed by western blotting.

### Mass spectrometric analysis of tPARP1-binding partners

To capture tPARP1-interacting proteins, we generated an SFB-tagged tPARP1 catalytic-dead mutant stable cell line. Twenty 10-cm dishes of SFB-mtPARP1 293T cells were harvested and the pellets were washed twice with ice-cold PBS followed by lysing in the NETN100. Cell lysates were cleaned by centrifugation for 5 min at 18,000× *g* at 4 °C. The soluble fragments were incubated with streptavidin beads for 2 h at 4 °C. Biotin was used to elute the protein complex and S-beads were incubated with the eluted solution for another 2 h at 4 °C. After four washes, the captured proteins were boiled in the 2× SDS loading buffer and subjected to the SDS-PAGE gel. The gel was recovered after the sample running into the separate gel. The sample was sent to the Taplin Biological Mass Spectrometry Facility at Harvard Medical School.

Excised gel bands were cut into approximately 1-mm^3^ pieces. Gel pieces were then subjected to a modified in-gel trypsin digestion procedure. Gel pieces were washed and dehydrated with acetonitrile for 10 min followed by removal of acetonitrile. Pieces were then completely dried in a speed-vacuum. The gel pieces was rehydrated with 50 mM ammonium bicarbonate solution containing 12.5 ng/µL modified sequencing-grade trypsin (Promega, Madison, WI) at 4 °C. After 45 min, the excess trypsin solution was removed and replaced with 50 mM ammonium bicarbonate solution to just cover the gel pieces. Samples were then placed at 37 °C overnight. Peptides were later extracted by removing the ammonium bicarbonate solution, followed by one wash with a solution containing 50% acetonitrile and 1% formic acid. The extracts were then dried in a speed-vacuum (~1 h). The samples were then stored at 4 °C until analysis.

On the day of analysis, the samples were reconstituted in 5–10 µL of high-performance liquid chromatography (HPLC) solvent A (2.5% acetonitrile, 0.1% formic acid). A nano-scale reverse-phase HPLC capillary column was created by packing 2.6 µm C18 spherical silica beads into a fused silica capillary (100 µm inner diameter × ~30 cm length) with a flame-drawn tip. After equilibrating the column, each sample was loaded via a Famos auto sampler (LC Packings, San Francisco, CA) onto the column. A gradient was formed and peptides were eluted with increasing concentrations of solvent B (97.5% acetonitrile, 0.1% formic acid).

As peptides eluted, they were subjected to electrospray ionization and then entered into an LTQ Orbitrap Velos Pro ion-trap mass spectrometer (Thermo Fisher Scientific, Waltham, MA). Peptides were detected, isolated, and fragmented to produce a tandem mass spectrum of specific fragment ions for each peptide. Peptide sequences (and hence protein identity) were determined by matching protein databases with the acquired fragmentation pattern by the software program, Sequest (Thermo Fisher Scientific, Waltham, MA). All databases include a reversed version of all the sequences and the data were filtered to a 1%–2% peptide false discovery rate. Data are available via ProteomeXchange with the identifier PXD018691.

### Cell apoptosis analysis

Apoptosis was analyzed by Annexin V-FITC binding and PI staining by flow cytometry. Briefly, U2OS cells, PARP1 knockout U2OS cells, or PARP1 D214N U2OS cells were harvested after 5 μg/mL poly(dA-dT) stimulation at the indicated time points. Cells were re-suspended with the Annexin V binding buffer, then Annexin V-FITC and PI were added and incubated at room temperature for 10 min in the dark. The samples were subjected to the flow cytometry.

### DeADP-ribosylation assays

The cells were lysed in NETN100 buffer containing protease inhibitor cocktails. Supernatants were collected by centrifugation for 5 min at 13,000 rpm and incubated with anti-ADPr antibody and beads for 2 h at 4 °C. To exclude the possibility that the modification on Pol III is poly-ADP-ribosylation, the immune-complex was used as a substrate for de-ADP-ribosylation reaction by supplementing with 2 μM PARG or 2 μM TARG1 to digest the PAR chain at 30 °C for 30 min before resolving in the SDS-PAGE for western blotting.

### IRF3 dimerization assay

The cells were collected at the indicated time point and lysed in the TBS lysis buffer (20 mM Tris, pH 7.5, 150 mM NaCl, and 0.5% NP-40) for IRF3 dimerization assay by western blotting using native SDS-PAGE. For the common western blotting assay, the NETN100 buffer was used as lysis buffer to analyze phosphorylation of IRF3.

### RT-qPCR analysis

The cells were stimulated with or without 5 μg/mL poly(dA-dT) at the indicated time point, and total RNA was isolated by TRIzol (Invitrogen) from cells according to the manufacturer’s instructions. Reverse transcription was performed using RNA samples to synthesize complementary DNA (cDNA) using a SuperScript III First-Strand Synthesis Kit (Invitrogen). The real-time PCR reactions were performed using the SYBR Green master mix (Bio-Rad) and with the optimized primer pairs (Supplementary Table S3) on a CFX Connect Real-Time PCR Detection System from Bio-Rad. Housekeeping gene *GAPDH* was employed for normalization.

### Virus infection

The Raw264.7 cells were infected with an engineered adenovirus type 5 (Ad-CMV-GFP from Vector Biolabs; this virus lacks the *E1* and *E3* genes) at 50 multiplicity of infection when 70%–80% confluence had been reached. DMEM supplemented with no serum was used for adenovirus infection. Cells were incubated with virus for 48 or 72 h, with occasionally gentle agitation. The virus was removed, and the cells were placed in complete medium until they were harvested.

### Preparation of nuclear and cytoplasmic fragments

The cells were lysed in buffer (10 mM HEPES, pH 7.5, 10 mM KCl, and 1.5 mM MgCl_2_) by douncing and centrifuged at 300 rpm for 5 min to remove cell debris. The supernatant was centrifuged at 1500 rpm for 5 min to collect the nuclear pellet. The supernatant was further centrifuged at 13,000 rpm for 10 min to collect the cytosolic supernatant.

### RNA interference

siRNA oligos at a final concentration of 40 nM were transfected into cells using Lipofectamine RNAimax reagent (Thermo Fisher Scientific) according to the manufacturer’s protocol. The transfection procedure using 20 nM siRNA was repeated on the next day. The cells were harvested on the third day for further analysis. siRNA targeting sequence for PARP9 was 5′-CCUUACCUUGGGUGAACUAAC-3′.

### Co-immunoprecipitation, western blot, and pull-down assay

Cells were lysed in NETN100 buffer containing protease inhibitor cocktails. Supernatants were collected by centrifugation for 5 min at 13,000 rpm and incubated with antibody and beads for 2 h at 4 °C. The immune-complex was washed followed by resolving in the SDS-PAGE for western blot analysis.

To test whether the ADP-ribosylation of Pol III is covalent or non-covalent, extraction of ADP-ribosylated proteins for immunoprecipitation analyses were modified as described in a previous study^68^. Briefly, cells were lysed in SDS lysis buffer (1% SDS, 10 mM HEPES, pH 7.0, 2 mM MgCl_2_, 500 U Benzonase, pH 8.5). Lysates were diluted with PBS to 0.2% SDS and mixed with antibody and beads for 2 h at 4 °C. Then the beads were washed with the SDS wash buffer (1% SDS, 100 mM HEPES, pH 8.5, 150 mM NaCl) and subsequently with the wash buffer (100 mM HEPES, pH 8.5, 150 mM NaCl). The ADP-ribosylated proteins were incubated with 2× SDS-PAGE sample loading buffer at 95 °C for 5 min and the eluates were subjected to immunoblotting analyses. To further confirm that ADP-ribosylation of Pol III is covalent, the 0.5 M NH_2_OH treatment at room temperature on an end-to-end rotator was performed to detach PAR chain from Pol III proteins before signal detection.

### Luciferase reporter assay

The reporter plasmid p125-Luc (p-IFN-β-Luc) was a gift from Dr. K. Fitzgerald in the University of Massachusetts Medical School. The Luciferase Reporter Assay Detection Kit and internal control plasmid pRL-TK was purchased from Promega. The cells were transfected using Lipofectamine 2000, according to the manufacturer’s instructions. For luciferase reporter assay with poly(dA-dT) stimulation, the cells in 24-well plates were co-transfected with 250 ng reporter plasmid and 50 ng internal control plasmid. Twenty four hours after transfection, cells were stimulated with 5 μg/mL poly(dA-dT) for the indicated time course. Each sample was performed in replicates and three independent experiments were set up. Ratios of firefly luciferase activity/renilla luciferase activity were normalized to the unstimulated group. Fold change values were determined.

### Purification of Pol III complex and in vitro transcription

The protocol for intercellular Pol III superfamily complex extraction was as described previously^50^. Briefly, to obtain S100, the cells were lysed in NETN100 buffer by homogeneously douncing and purified by centrifugation. The Pol III complex was captured by FLAG-M2 beads (Sigma) at 4 °C. After four times harsh washing, Pol III protein with FLAG tag was eluted from the resin with 100 µg/mL FLAG peptide (Sigma). Eluted fractions were pooled and dialyzed against PBS for overnight. Then the eluate was purified by POLR3G antibody for another 1 h. The bound protein complex was washed and stored for the in vitro transcription assay. Pol III in vitro transcription reaction contained Pol III transcription buffer (1 μM DTT, 10 μg/mL alpha amanatin, 1 U RNase inhibitor, 50 μM TTP, 0.15 μM ^32^P-UTP, and 250 ng poly(dA-dT)). RNA samples were separated on 6% denaturing polyacrylamide gel containing 8 M of urea and detected by autoradiography.

### Statistical analysis

Data are represented as means ± SD as indicated from three independent experiments. Significance of differences was evaluated by Student’s *t*-test. N.S., no significance; *P < 0.05; **P < 0.01; ***P < 0.001.

## Supplementary information

Supplementary Information

## Data Availability

The mass spectrometry proteomics data have been deposited to the ProteomeXchange Consortium via the PRIDE^69^ partner repository with the data set identifier PXD018691. The additional data that support the findings of this study are available from the corresponding author on request.
