# Supplementary material for: Truncated PARP1 mediates ADP-ribosylation of RNA polymerase III for apoptosis
Source: Cell Discov. 2022 Jan 18;8:3. doi: 10.1038/s41421-021-00355-1 (PMC8764063; doi:10.1038/s41421-021-00355-1)
Supplement: Supplementary file 1 — Supplementary Information [file 41421_2021_355_MOESM1_ESM.pdf]

Supplementary Information

Truncated PARP1 promotes RNA polymerase III during cytosolic DNA induced-apoptosis

Qian Chen[#]*, Kai Ma[#], Xiuhua Liu, Shih-Hsun Chen, Peng Li, Yonghao Yu, Anthony K. L. Leung, Xiaochun Yu*

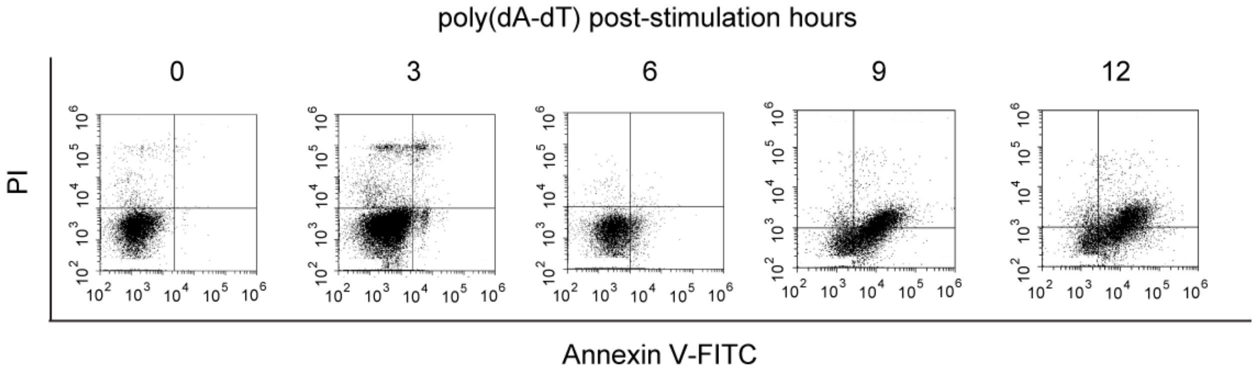

Supplementary Fig. S1. The flow cytometry images of apoptosis-induced by 5 μg/ml of poly(dA-dT) in U2OS cells.

a

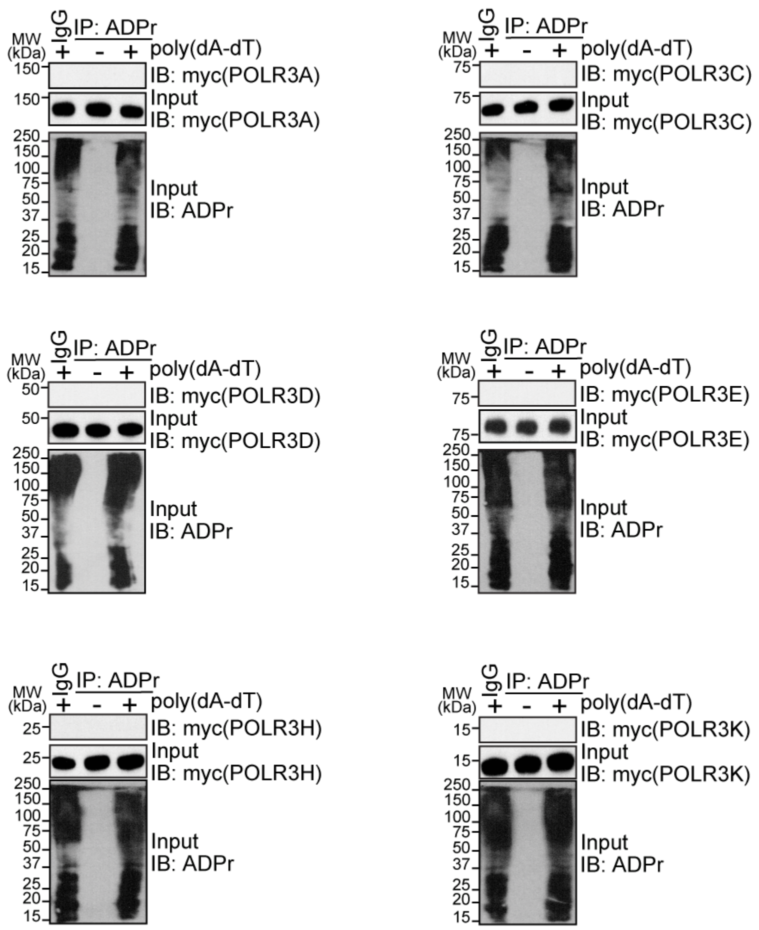

b

PolⅢ subunit	MW(kDa)	Function	ADP-ribosylation
POLR3A	155.6	Active complex	-
POLR3B	127.6	Active complex	+
POLR3C	60.5	Initiation complex	-
POLR3D	44.4	Termination complex	-
POLR3E	79.8	Termination complex	-
POLR3F	35.6	Initiation complex	+
POLR3G	25.9	Initiation complex	+
POLR3H	22.9	Initiation complex	-
POLR3K	12.3	Termination complex	-

Supplementary Fig. S2. ADP-ribosylation of POLR3A, POLR3C, POLR3D, POLR3E, POLR3H and POLR3K are not detectable during poly(dA-dT)-stimulated apoptosis. (a) Individual subunit of Pol III with a myc tag was expressed in the cells, followed by poly(dA-dT)-stimulated apoptosis (5 μg/ml, six hours). IP and Western blot were performed with anti-ADP-ribose antibody and anti-myc antibody respectively. (b) Summary of the ADP-ribosylated RNA Pol III subunits during the poly(dA-dT)-stimulated apoptosis.

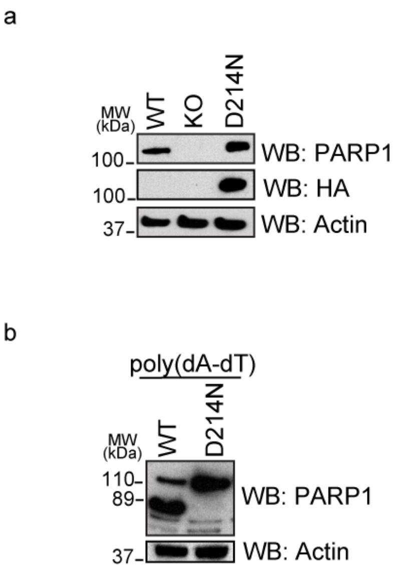

Supplementary Fig. S3. The expression of caspase-resistant PARP1 (D214N) in PARP1-null cells. (a) Indicated antibodies were used in the Western blot to confirm the expression of the D214N mutant of PARP1. (b) PARP1 cleavage was examined in the poly(dA-dT)-stimulated cells (5 μg/ml, six hours). D214N could not be cut by caspases during apoptosis.

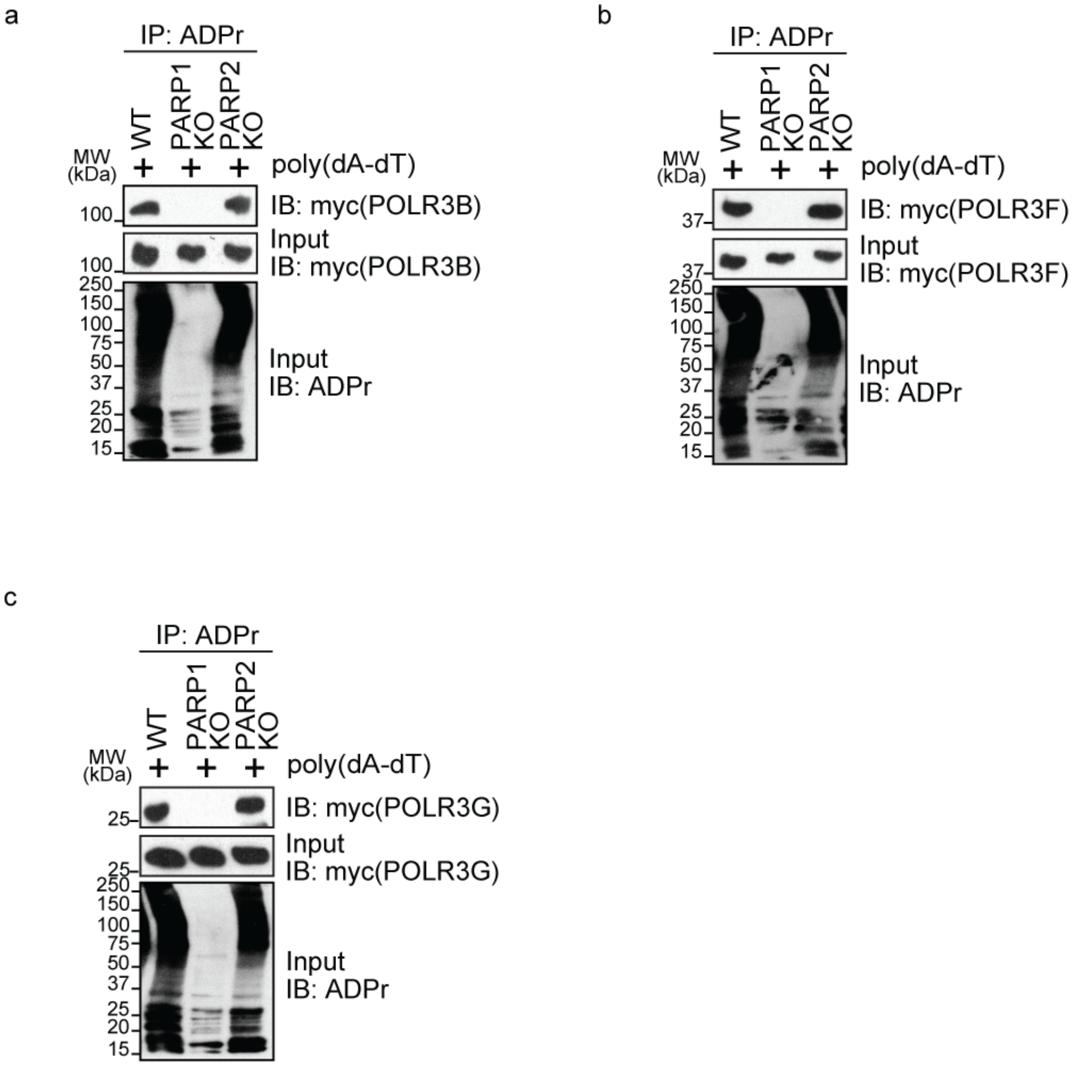

Supplementary Fig. S4. PARP1 but not PARP2 is required for the ADP-ribosylation of Pol III during apoptosis. (a, b and c) Myc-tagged Pol III B, F or G subunit was expressed in the wide type, PARP1-null or PARP2-null U2OS cells which were stimulated by 5 µg/ml of poly(dA-dT) for six hours. The ADP-ribosylated Pol III was IPed with anti-ADPr antibody from the lysates of wide type, PARP1-null or PARP2-null cells and examined by Western blotting with anti-myc antibody.

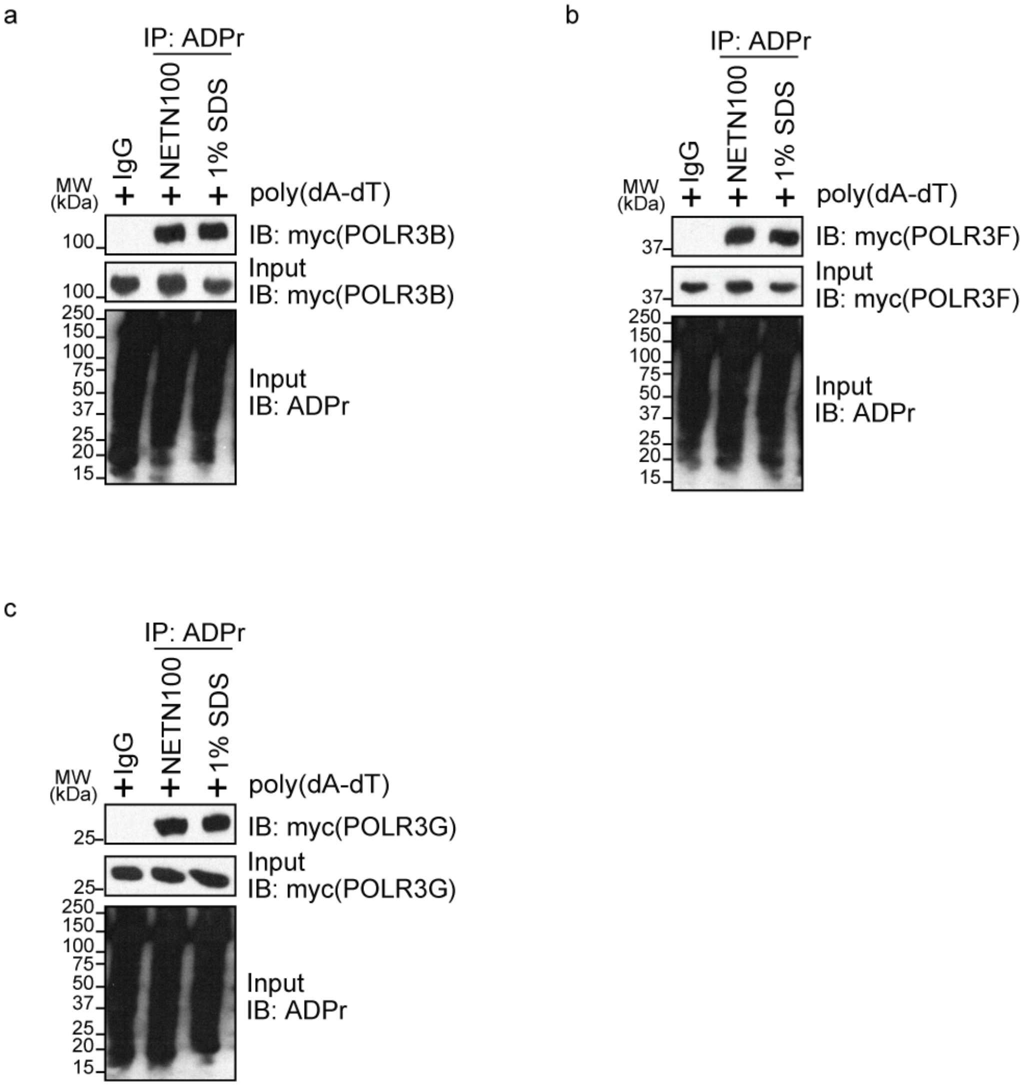

Supplementary Fig. S5. ADP-ribosylation on Pol III is a covalent modification. (a, b and c) Myc-tagged Pol III B, F or G subunit was expressed in the poly(dA-dT)-stimulated U2OS cells (5 μg/ml, six hours), which were lyzed by NETN100 buffer to retain non-covalent interaction in cell lysates or 1% SDS buffer to abolish non-covalent interaction in cell lysates. The samples were examined by IPed with anti-ADPr antibody and Western blotting with anti-myc antibody.

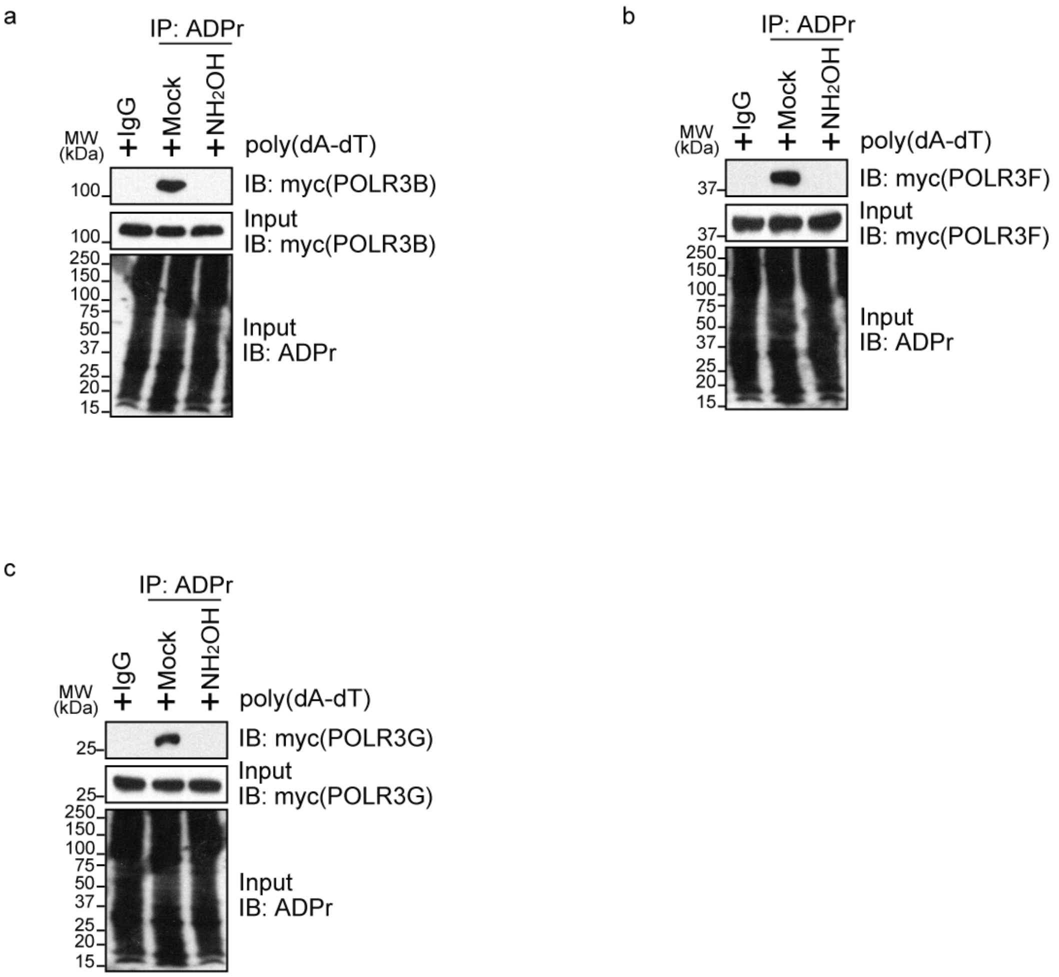

Supplementary Fig. S6. Covalent ADP-ribosylation on Pol III is validated by NH_2OH treatment. (a, b and c) Myc-tagged Pol III B, F or G subunit was expressed in the poly(dA-dT)-stimulated U2OS cells (5 μg/ml, six hours), which were lyzed by NETN100 buffer. The ADP-ribosylated proteins were IPed with anti-ADPr antibody and followed by NH_2OH treatment (0.5 M) to remove covalently linked ADPr, and the samples were further examined by Western blotting using anti-myc antibody.

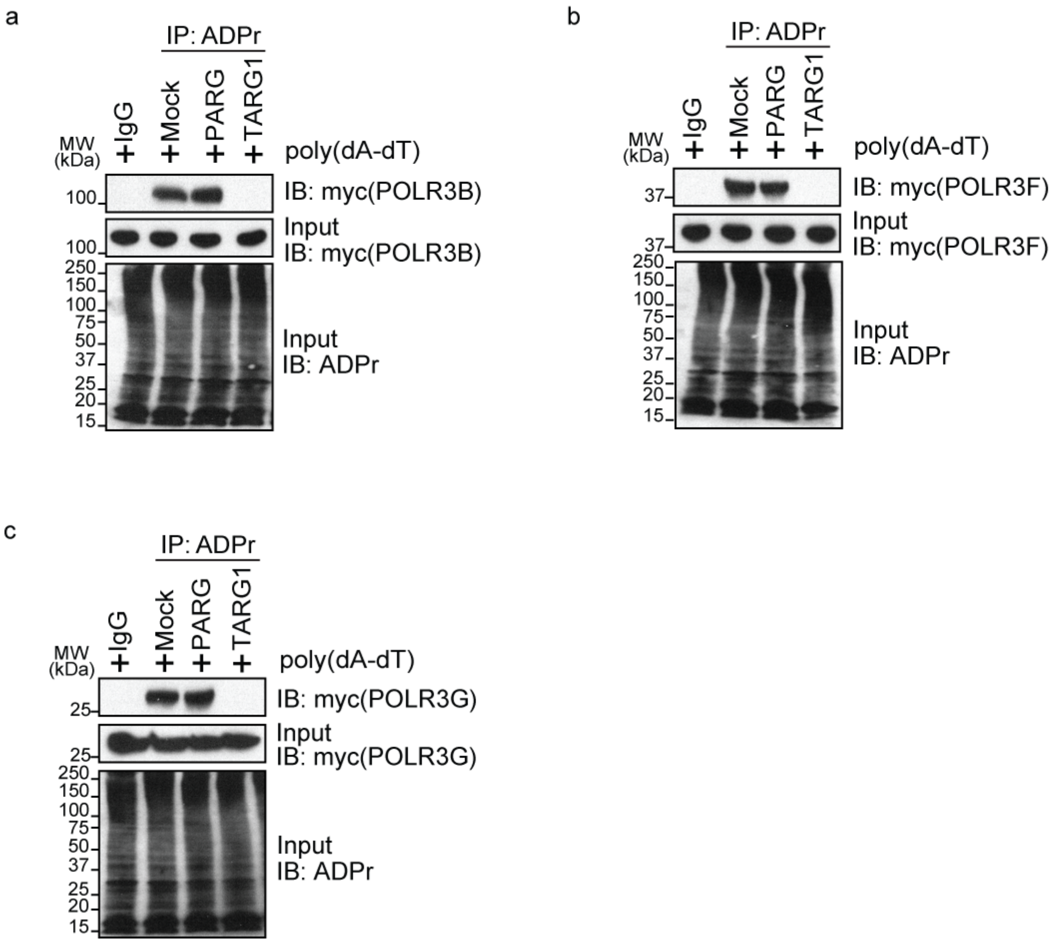

Supplementary Fig. S7. PARG obviously cannot remove the ADP-ribosylation on Pol III. (a, b and c) Myc-tagged Pol III B, F or G subunit was expressed in the poly(dA-dT)-stimulated U2OS cells (5 μg/ml, six hours). The ADP-ribosylated Pol III was IPed with anti-ADPr antibody and the IPed complex was treated with PARG or TARG1. The samples were examined by Western blotting.

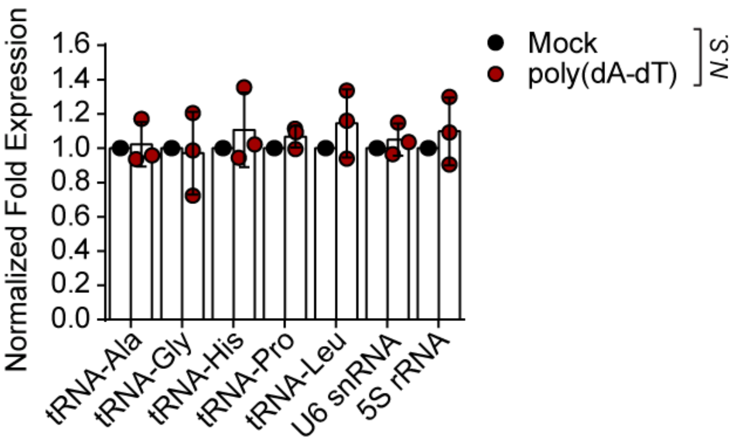

Supplementary Fig. S8. ADP-ribosylation on Pol III does not affect the expression of Pol III-dependent RNA transcription during the early phase of apoptosis. Five hours after treatment with 5 μg/ml of poly(dA-dT), the expression of several tRNAs, U6 snRNA or 5S rRNA was analyzed by RT-qPCR in U2OS cells. Data are represented as mean ± s.d. as indicated from three independent experiments. Significance of differences was evaluated by Student's t test. *N.S.*: Non-Significant.

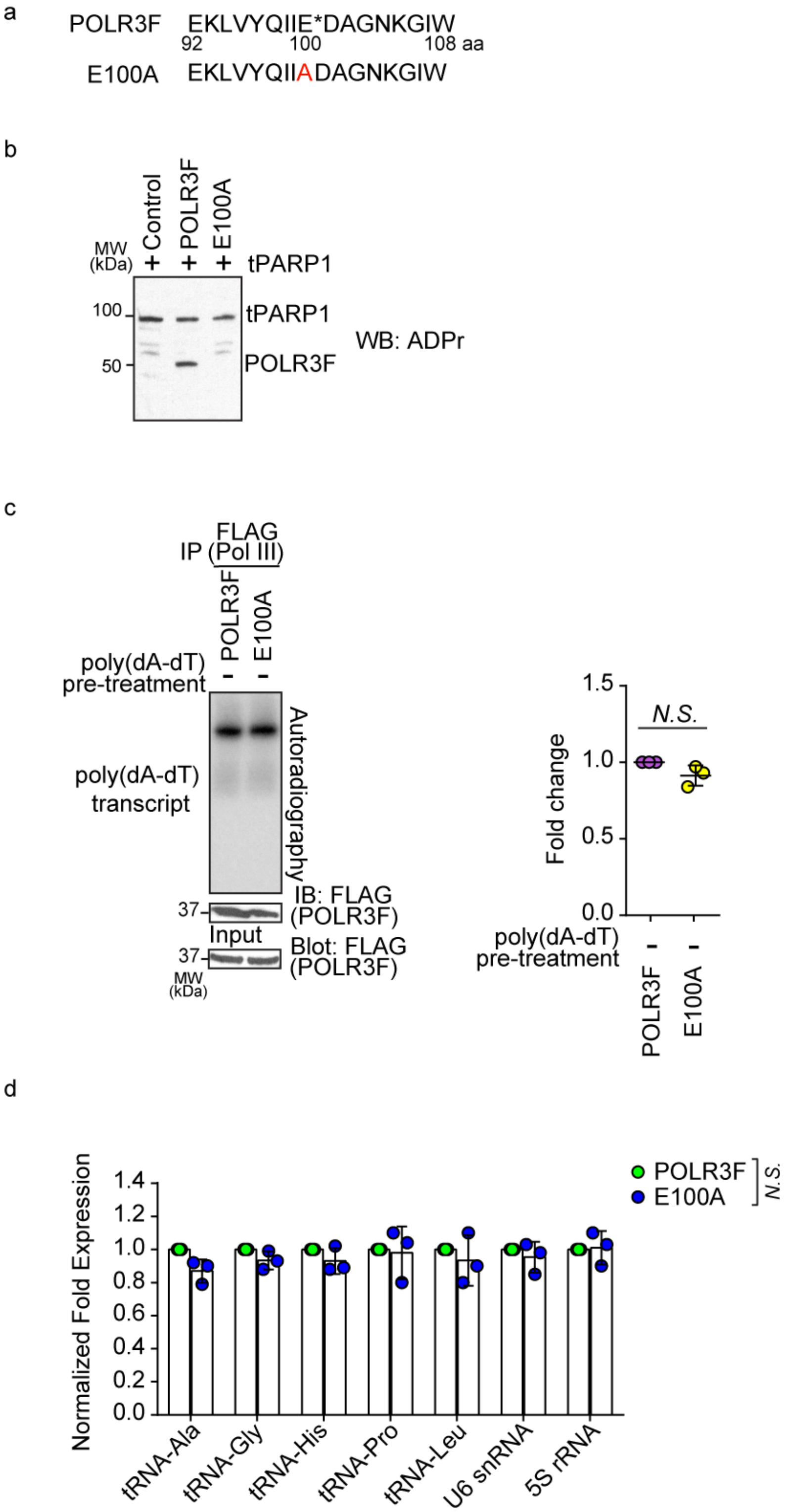

Supplementary Fig. S9. E100 of POLR3F does not impair the enzymatic activity of Pol III without poly(dA-dT) treatment. (a) The amino acid sequence (92-108 aa) of POLR3F is shown,

and asterisk denotes E100 in POLR3F. E100A was generated by replacing E100 with Ala. (b) E100A was unable to be ADP-ribosylated by tPARP1 *in vitro*. (c) The enzymatic activity of E100A mutation on poly(dA-dT) transcripts was similar with wide type without poly(dA-dT) treatment. (d) No obvious changes were observed in the transcriptions of general Pol III targets in E100A mutation without poly(dA-dT) treatment. Data are represented as mean ± s.d. as indicated from three independent experiments. Significance of differences was evaluated by Student's t test. *N.S.*: Non-Significant.

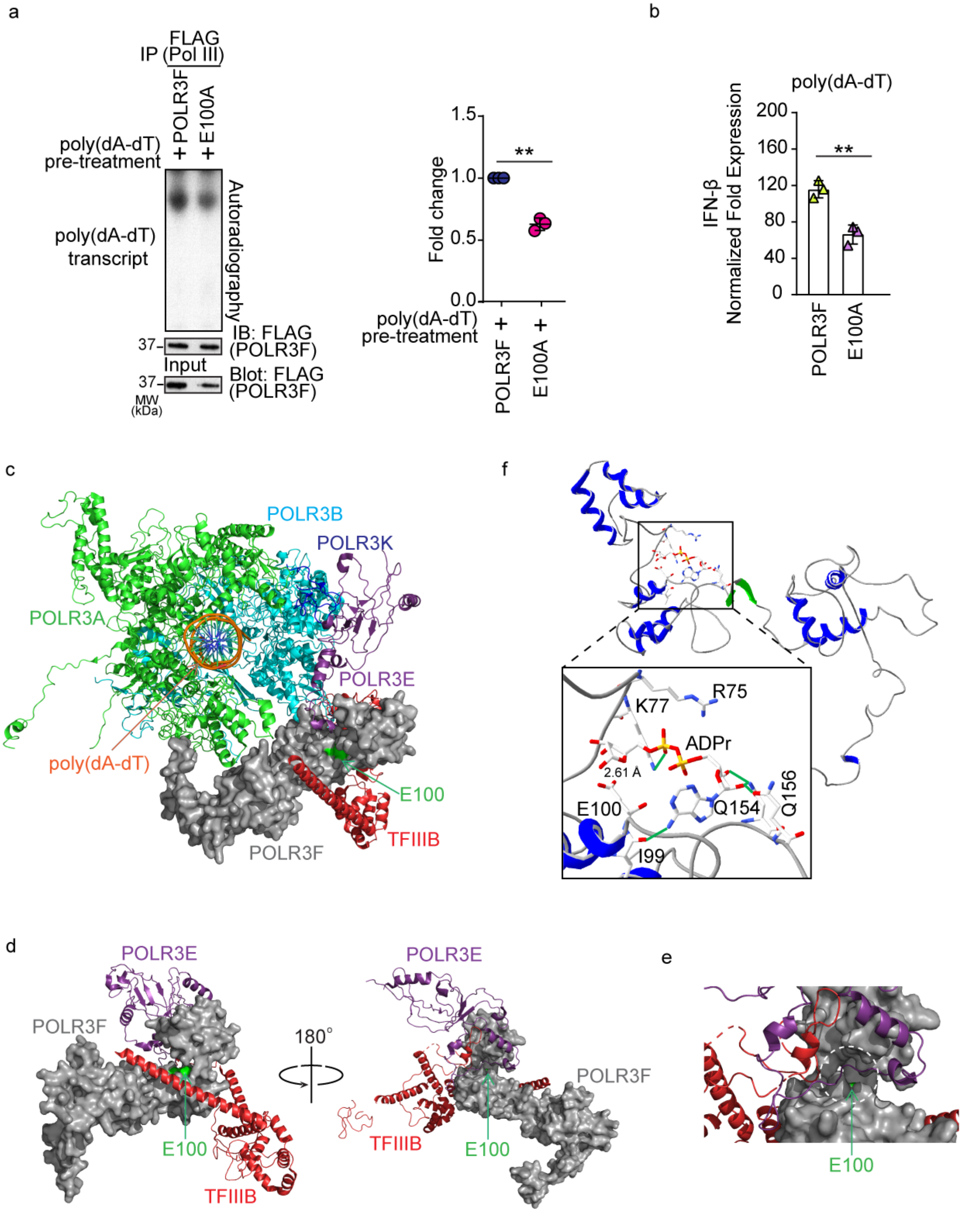

Supplementary Fig. S10. E100 of POLR3F regulates the enzymatic activity of Pol III during

poly(dA-dT)-stimulated apoptosis. (a) The E100A mutation of POLR3F reduces the enzyme activity of Pol III after poly(dA-dT) treatment (5 μg/ml, six hours). (b) The E100A mutation of POLR3F inhibited the IFN-β induction after poly(dA-dT) treatment (5 μg/ml, six hours). (c) Model of ADP-ribosylated POLR3F regulates the activity of Pol III. The Pol III subunits POLR3A-POLR3B-POLR3E-POLR3F-POLR3K and TFIII B are individually colored. poly(dA-dT) and E100 in POLR3F are depicted in orange and green, respectively. (d) Structure of the POLR3E-POLR3F-TFIII B complex. E100 of POLR3F is in a flexible pocket. (e) The pocket of POLR3F was circled by dashed line. (f) The ADP-ribosylation of E100 of POLR3F is modeled by GEMDOCK. The docked pose is located in the pocket mainly formed by K77, R75, I99, E100, Q154, and Q156. Green and black dashes indicate possible hydrogen bonds and the distance between two atoms, respectively. Data are represented as mean ± s.d. as indicated from three independent experiments. Significance of differences was evaluated by Student's t test. **: Statistically Significant ($p < 0.01$).

a

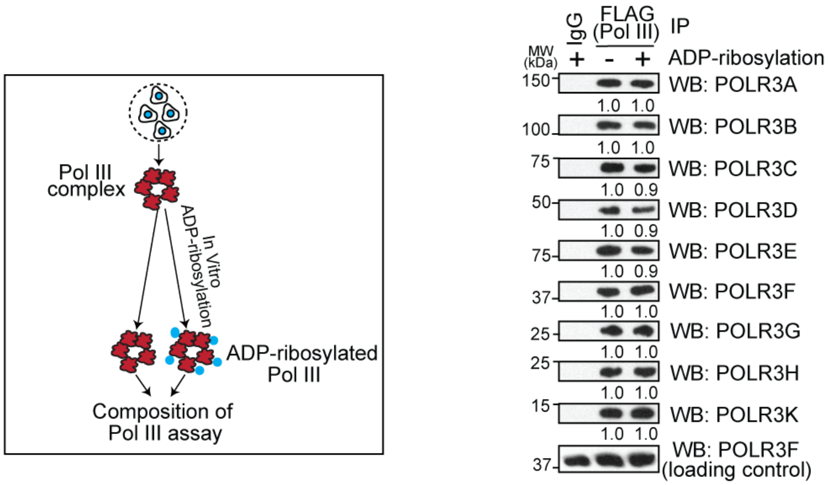

b

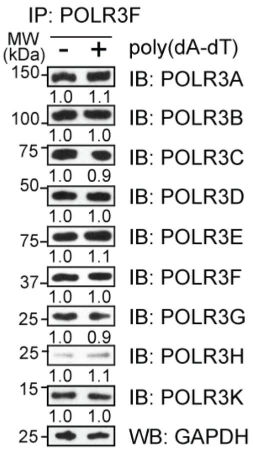

Supplementary Fig. S11. ADP-ribosylation does not alter the composition of Pol III complex. (a) Pol III complex was purified from U2OS cells stably expressing FLAG-POLR3F and ADP-ribosylated by tPARP1 *in vitro*. The components of ADP-ribosylated Pol III complex were examined by Western blot with specific antibodies against individual subunit in the Pol III complex. The expression of POLR3F was used as a loading control. (b) Pol III complex was IPed from cytoplasmic fraction in U2OS cells treated with or without poly(dA-dT) (5 µg/ml, six hours). The Pol III complex was examined with indicated antibodies.

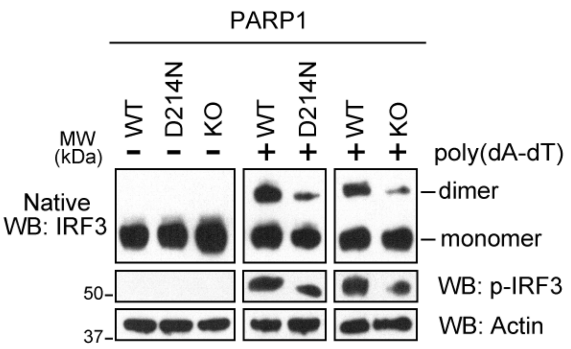

Supplementary Fig. S12. PARP1 cleavage promotes RIG-I activation during poly(dA-dT)-induced apoptosis. IRF3 dimerization and phosphorylation of IRF3 were analyzed in wide type U2OS cells, PARP1-null cells or non-cleavable PARP1 cells (D214N) with or without poly(dA-dT) (5 µg/ml, six hours) using Western blotting with specific antibodies.

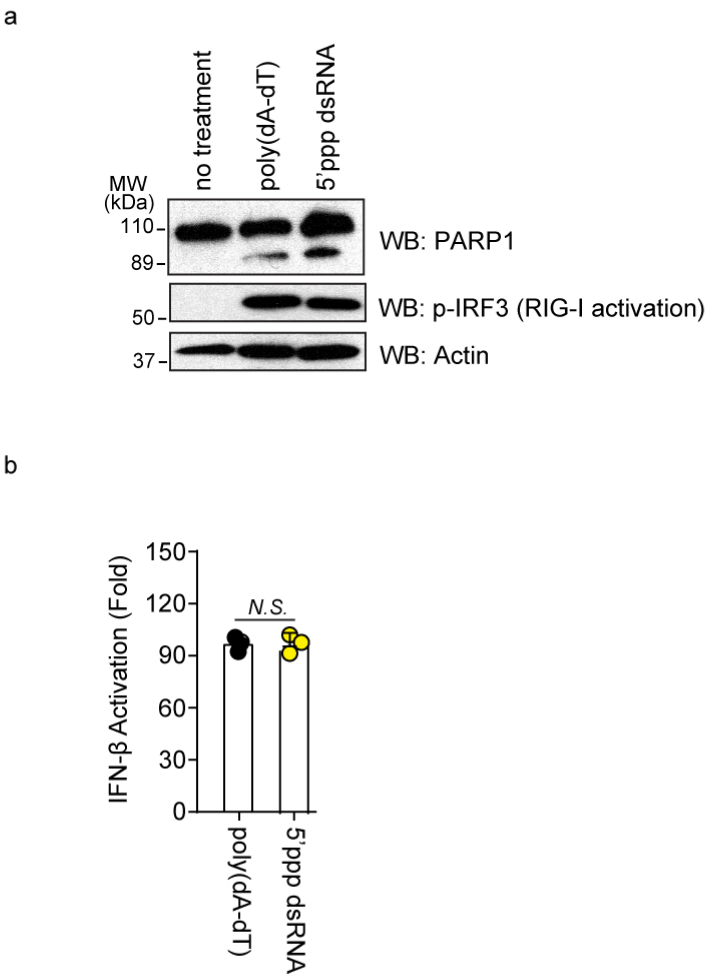

Supplementary Fig. S13. Apoptotic cell response to the direct RIG-I stimulation. (a) PARP1 cleavage and RIG-I activation were examined in wild-type U2OS cells after 5 μg/ml of poly(dA-dT) or 5' triphosphate double-stranded RNA (5'ppp dsRNA) induction. (b) Induction of IFN-β by 5 μg/ml of poly(dA-dT) or 5' triphosphate double-stranded RNA (5'ppp dsRNA) was analyzed by luciferase reporter assay in wild-type U2OS cells. Data are represented as mean ± s.d. as indicated from three independent experiments. Significance of differences was evaluated by Student's t test. *N.S.*: Non-Significant.

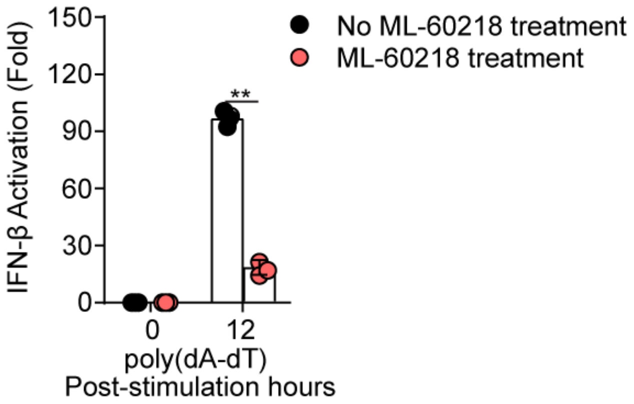

Supplementary Fig. S14. tPARP1 facilitates the generation of IFN-β through Pol III transcription in poly(dA-dT)-mediated apoptotic cells. The cells were treated with 30 μM Pol III inhibitor ML-60218 for 2 hours to inhibit the transcription activity of Pol III, then the IFN-β production was examined by luciferase reporter assay. Data are represented as mean ± s.d. as indicated from three independent experiments. Significance of differences was evaluated by Student's t test. **: Statistically Significant ($p < 0.01$).

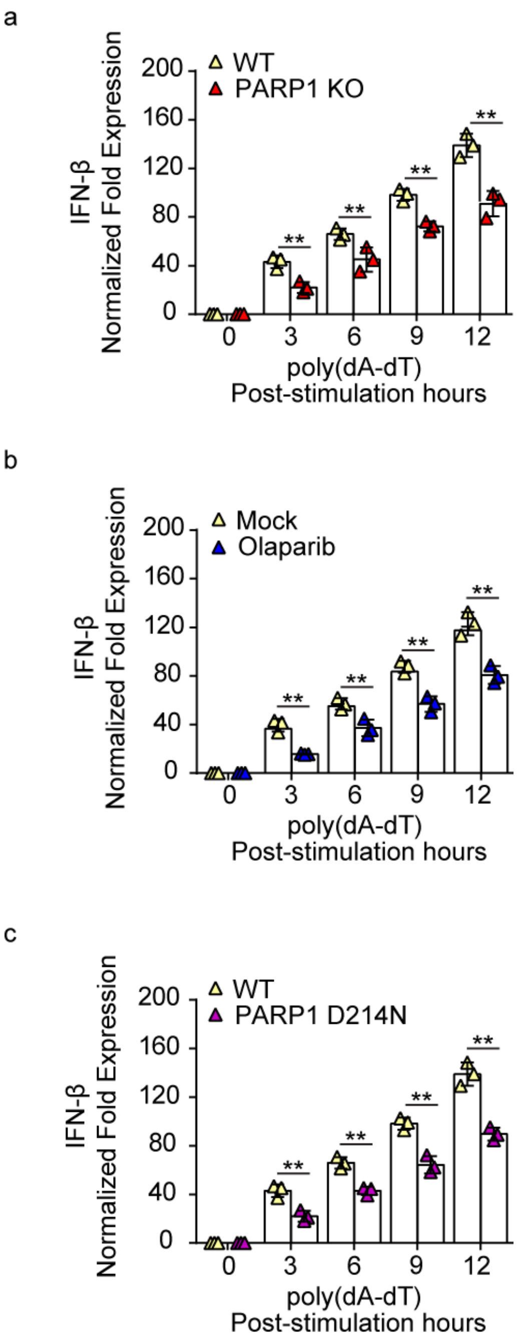

Supplementary Fig. S15. PARP1 cleavage promotes the production of IFN-β during poly(dA-dT)-induced apoptosis through RT-qPCR analysis. (a) Induction of IFN-β by poly(dA-dT) was analyzed by RT-qPCR in wide type U2OS cells or PARP1-null cells. (b) Induction of IFN-β by poly(dA-dT) was examined by RT-qPCR in U2OS cells pre-treated with or without 1 μM olaparib

for 1 hour. (c) Induction of IFN-β by poly(dA-dT) in PARP1-null cells reconstructed with wild-type PARP1 or the D214N mutant was determined by RT-qPCR. Note that the cells were harvested after six hours post-stimulation by 5 μg/ml of poly(dA-dT). Data are represented as mean ± s.d. as indicated from three independent experiments. Significance of differences was evaluated by Student's t test. **: Statistically Significant ($p < 0.01$).

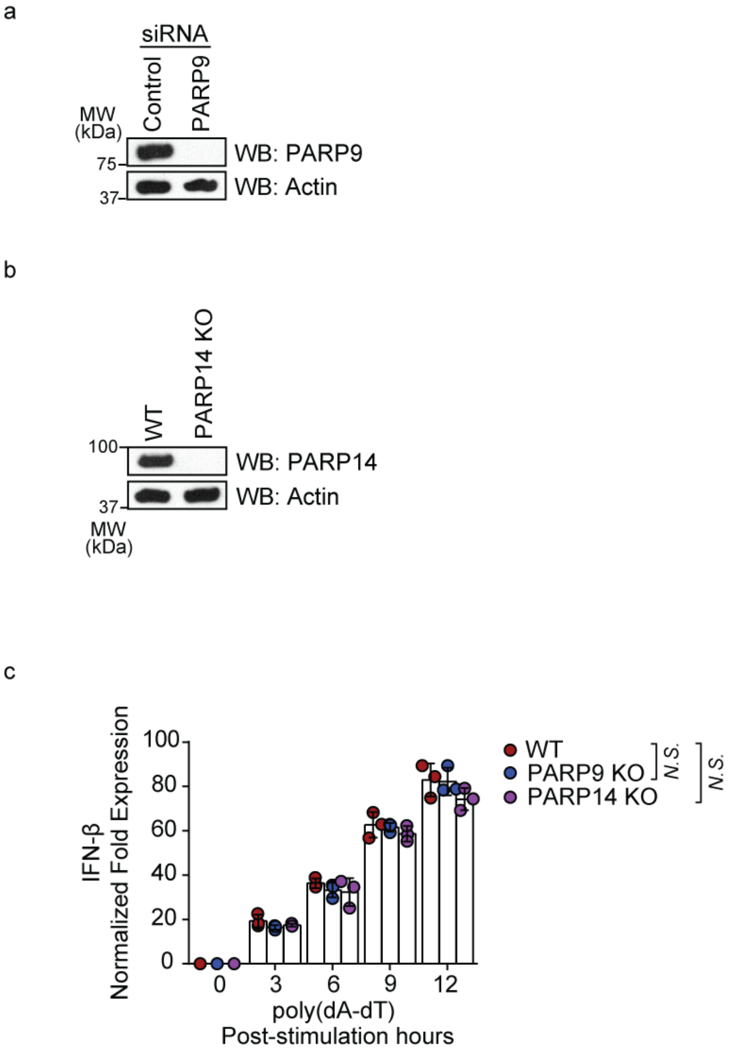

Supplementary Fig. S16. PARP9 or PARP14 has little impact on IFN-β expression during poly(dA-dT)-induced apoptosis. (a) A549 cells were treated with control siRNA or PARP9 siRNA. The expression level of PARP9 in the cells was examined by Western blot with anti-PARP9 antibody. (b) The depletion of PARP14 was confirmed by Western blot with anti-PARP14 antibody in wide type A549 cells and PARP14 KO cells. (c) Induction of IFN-β was examined by RT-qPCR in wild type cells, PARP9-null cells or PARP14-null cells treated with 5 μg/ml of poly(dA-dT) for six hours. Data are represented as mean ± s.d. as indicated from three independent experiments. Significance of differences was evaluated by Student's t test. *N.S.*: Non Significant.

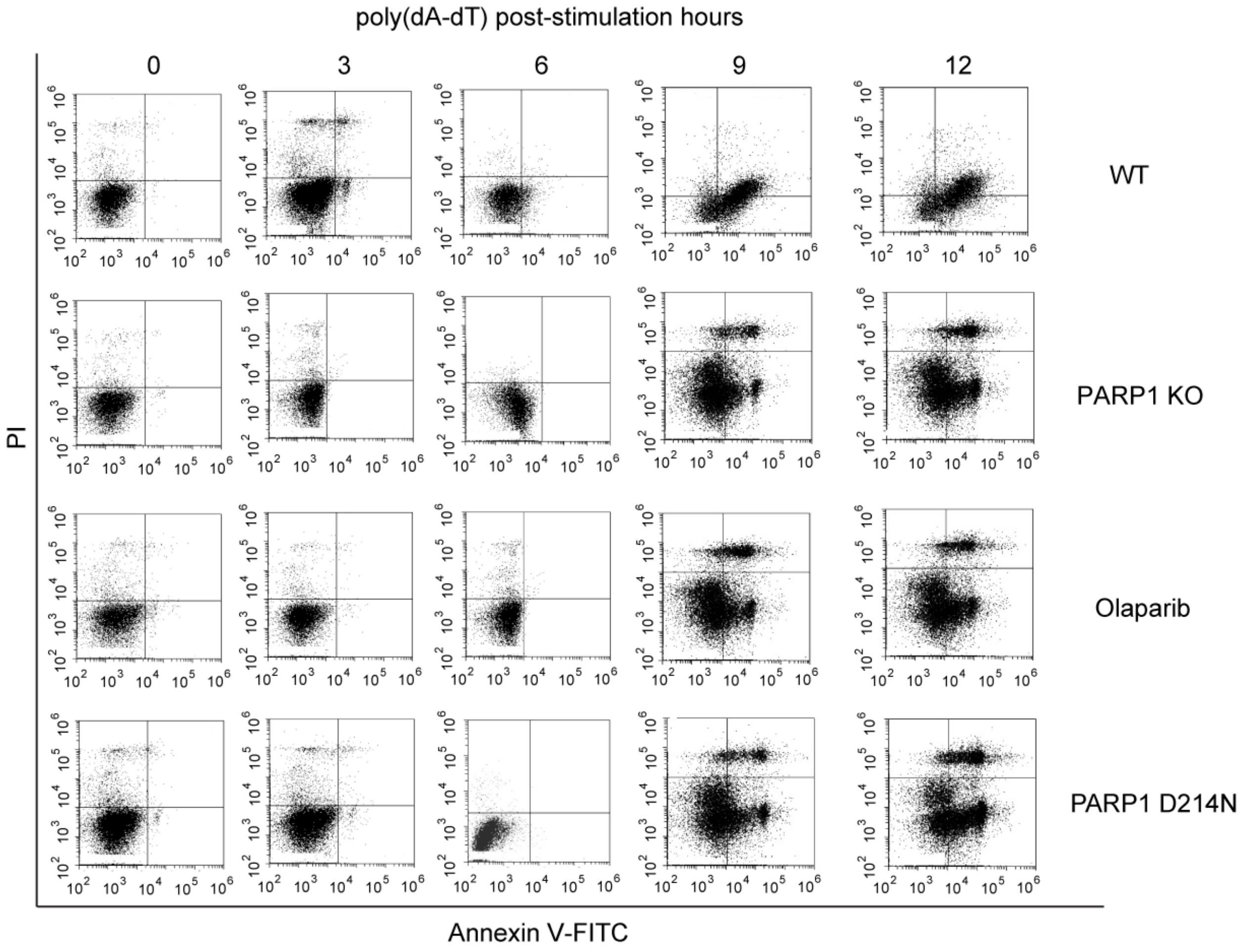

Supplementary Fig. S17. The flow cytometry images of apoptosis-induced by 5 µg/ml of poly(dA-dT) in wide-type U2OS cells, PARP1 KO U2OS cells, olaparib-treated cells and PARP1 D214N U2OS cells.

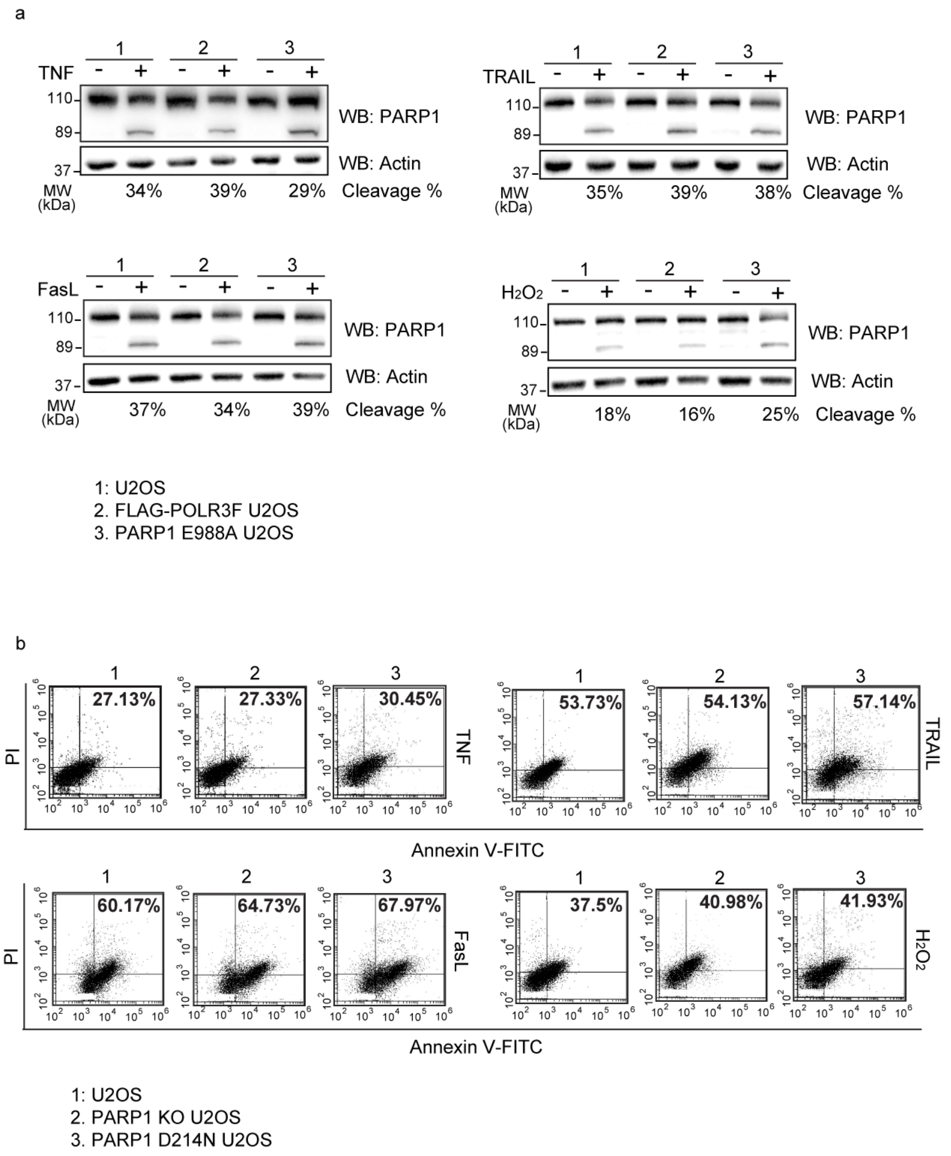

Supplementary Fig. S18. The parental and engineered cells in apoptosis triggered in TNF, TRAIL, FasL or H_2O_2. (a) The cleavage of PARP1 was examined in TNF-, TRAIL-, FasL- or H_2O_2-stimulated apoptotic U2OS cells, FLAG-POLR3F U2OS cells or PARP1 E988A U2OS cells. The ratio of cleaved PARP1 was included. (b) Apoptosis-induced by TNF, TRAIL, FasL or H_2O_2 in U2OS cells, PARP1 KO U2OS cells or PARP1 D214N U2OS cells was determined by Annexin V-FITC/PI double-staining.

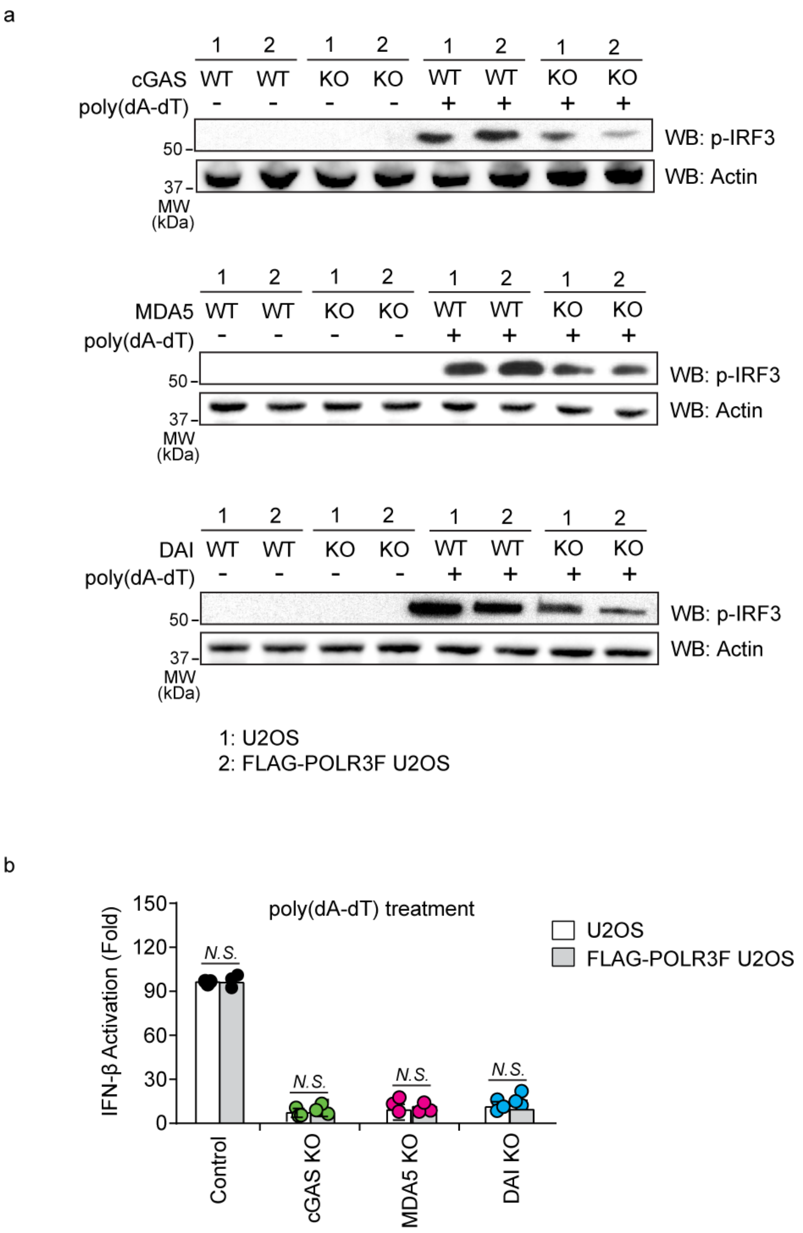

Supplementary Fig. S19. The activation of non-RIG-I PRR in FLAG-POLR3F U2OS cells. (a) The IRF3 phosphorylation in cGAS-, MDA5- and DAI-deficient cells after poly(dA-dT) treatment was detected using Western blotting. (b) Induction of IFN-β by poly(dA-dT) was analyzed by luciferase reporter assay in FLAG-POLR3F U2OS cells or cGAS-, MDA5- and DAI-null cells. Data are represented as mean ± s.d. as indicated from three independent experiments. Significance of differences was evaluated by Student's t test. N.S.: Non Significant.

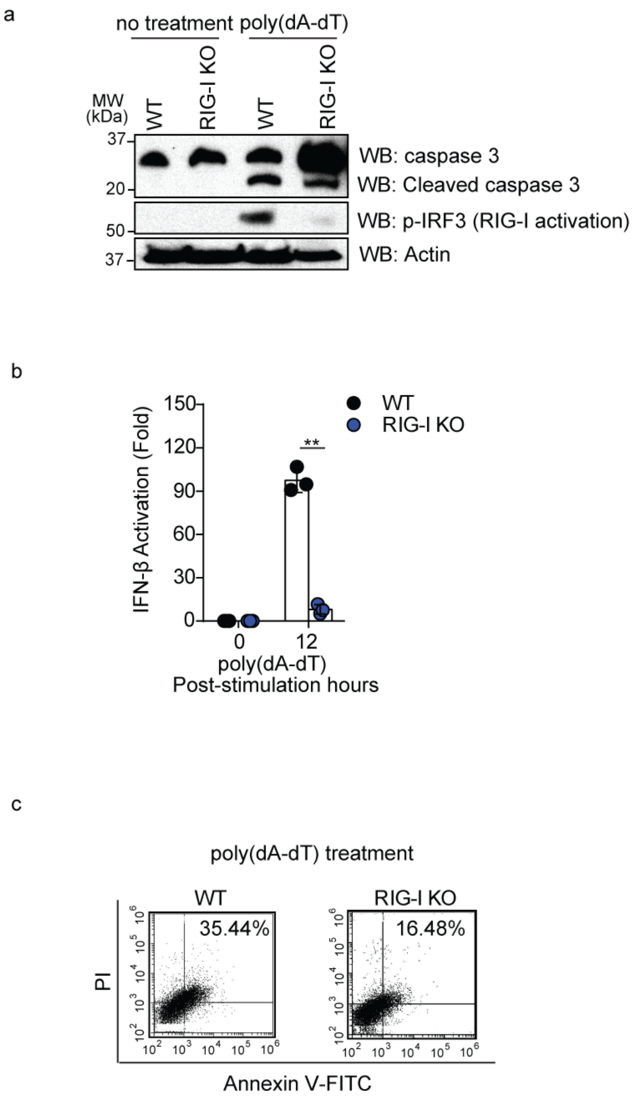

Supplementary Fig. S20. RIG-I pathway promotes poly(dA-dT)-induced apoptosis. (a) Caspase 3 cleavage and phosphorylation of IRF3 were analyzed in wide-type U2OS cells or RIG-I-null cells with poly(dA-dT) (5 μg/ml, six hours) using Western blotting with specific antibodies. (b) Induction of IFN-β by 5 μg/ml of poly(dA-dT) was analyzed by luciferase reporter assay in wild-type U2OS cells or RIG-I-null cells. (c) Apoptosis-induced by 5 μg/ml of poly(dA-dT) in wild-type U2OS cells or RIG-I-null cells was determined by Annexin V-FITC/PI double-staining. Data are represented as mean ± s.d. as indicated from three independent experiments. Significance of differences was evaluated by Student's t test. **: Statistically Significant ($p < 0.01$).

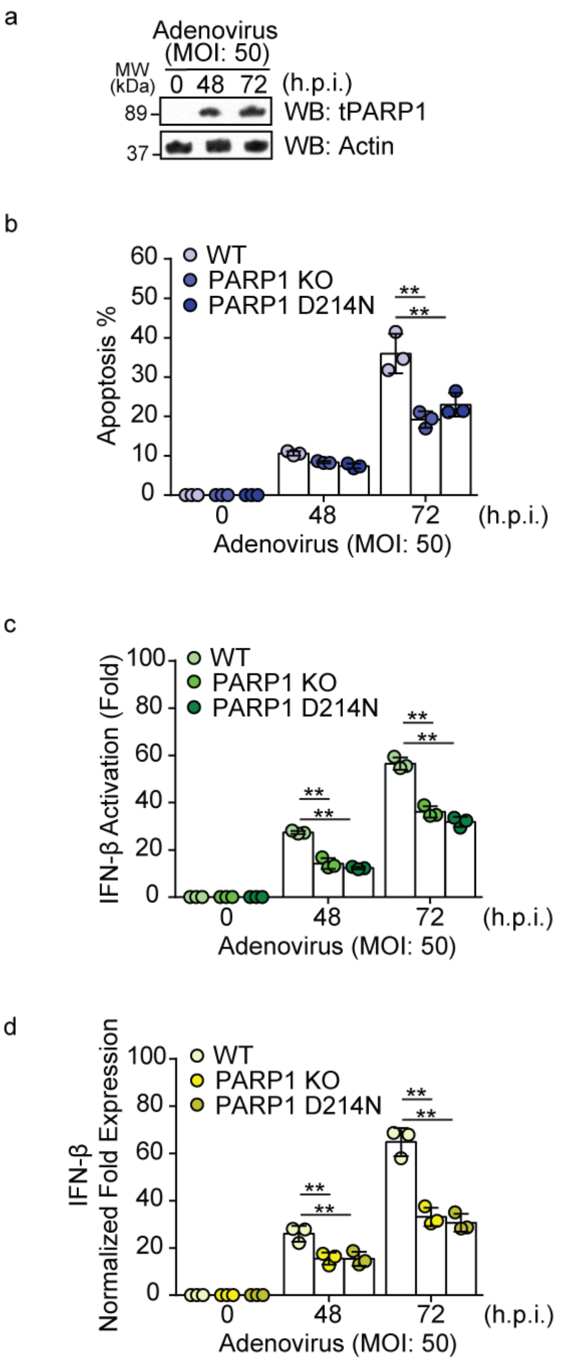

Supplementary Fig. S21. PARP1 cleavage facilitates innate immune response and promotes apoptosis induced by adenovirus infection. (a) Raw264.7 cells were infected with adenovirus at the 50 MOI with 48 hours or 72 hours. The cleavage of PARP1 was examined in a time course in adenovirus-induced apoptotic cells with anti-PARP1 C-terminus antibody. (b) Apoptosis-induced

by adenovirus in wide type Raw264.7 cells, PARP1-null cells or non-cleavable PARP1 cells (D214N) was determined by Annexin V-FITC/PI double-staining. (c) Induction of IFN-β by adenovirus was analyzed by luciferase reporter assay in wide type cells, PARP1-null cells or non-cleavable PARP1 cells (D214N). (d) Induction of endogenous IFN-β by adenovirus was analyzed by RT-qPCR in wide type cells, PARP1-null cells or non-cleavable PARP1 cells (D214N). Data are represented as mean ± s.d. as indicated from three independent experiments. Significance of differences was evaluated by Student's t test. **: Statistically Significant ($p < 0.01$).

Supplemental Table S1. Tandem affinity purification and mass spectrometry of mutant tPARP1 (mtPARP1).

Unique	Total	Reference	Gene Symbol	MWT(kDa)	AVG
109	377	P09874_PARP1_HUMAN	PARP1	113.01	2.5485
54	83	P12956_XRCC6_HUMAN	XRCC6	69.8	3.0713
53	86	P13010_XRCC5_HUMAN	XRCC5	82.65	2.7697
19	19	Q16531_DDB1_HUMAN	DDB1	126.89	2.4996
16	16	P11142_HSP7C_HUMAN	HSPA8	70.85	3.3824
15	17	P11021_GRP78_HUMAN	HSPA5	72.29	3.2708
13	13	P38646_GRP75_HUMAN	HSPA9	73.63	2.9679
12	12	P08107_HSP71_HUMAN	HSPA1A	70.01	2.9184
11	12	Q14527_HLTF_HUMAN	HLTF	113.86	2.7495
10	12	P62805_H4_HUMAN	HIST1H4A	11.36	2.8744
10	10	P23396_RS3_HUMAN	RPS3	26.67	2.8998
9	11	P34931_HS71L_HUMAN	HSPA1L	70.33	3.5281
8	9	Q13885_TBB2A_HUMAN	TUBB2A	49.87	3.2208
7	7	O15160_RPAC1_HUMAN	POLR1C	39.23	3.393
7	7	Q00059_TFAM_HUMAN	TFAM	29.08	2.4731
7	7	P27694_RFA1_HUMAN	RPA1	68.1	2.4104
6	7	Q96RQ3_MCCA_HUMAN	MCCC1	80.42	3.1271
6	6	Q71U36_TBA1A_HUMAN	TUBA1A	50.1	3.3291
6	6	O14802_RPC1_HUMAN	POLR3A	155.54	2.403
5	6	P33778_H2B1B_HUMAN	HIST1H2BB	13.94	2.9021

5	5	O60264_SMCA5_HUMAN	SMARCA5	121.83	3.437
5	5	P04279_SEMG1_HUMAN	SEMG1	52.1	3.0447
5	5	P07355_ANXA2_HUMAN	ANXA2	38.58	2.9969
5	5	Q13112_CAF1B_HUMAN	CHAF1B	61.45	2.6485
4	7	Q16695_H31T_HUMAN	HIST3H3	15.5	2.1025
4	5	Q04837_SSBP_HUMAN	SSBP1	17.25	3.4348
4	5	Q9UNY4_TTF2_HUMAN	TTF2	129.51	2.5979
4	4	P16220_CREB1_HUMAN	CREB1	36.67	3.4467
4	4	P33993_MCM7_HUMAN	MCM7	81.26	3.2773
4	4	P62987_RL40_HUMAN	UBA52	14.72	2.9192
4	4	P68104_EF1A1_HUMAN	EEF1A1	50.11	2.7684
4	4	P54652_HSP72_HUMAN	HSPA2	69.98	2.5195
4	4	Q9NW08_RPC2_HUMAN	POLR3B	127.7	2.4926
4	4	Q96QV6_H2A1A_HUMAN	HIST1H2AA	14.22	2.4777
4	4	P15927_RFA2_HUMAN	RPA2	29.23	2.3949
4	4	Q9H1D9_RPC6_HUMAN	POLR3F	35.66	2.3889
3	4	P10809_CH60_HUMAN	HSPD1	61.02	3.4653
3	4	P31260_HXA10_HUMAN	HOXA10	42.39	3.1152
3	4	Q13426_XRCC4_HUMAN	XRCC4	38.26	2.8689
3	4	P15336_ATF2_HUMAN	ATF2	54.5	2.7039
3	4	P07910_HNRPC_HUMAN	HNRNPC	33.65	2.5858
3	3	Q12800_TFCP2_HUMAN	TFCP2	57.22	4.1159
3	3	P05412_JUN_HUMAN	JUN	35.65	4.0784
3	3	P31943_HNRH1_HUMAN	HNRNPH1	49.2	3.7096
3	3	Q09028_RBBP4_HUMAN	RBBP4	47.63	3.4654

3	3	Q9UQR1_ZN148_HUMAN	ZNF148	88.92	3.1581
3	3	Q15517_CDSN_HUMAN	CDSN	51.49	3.1575
3	3	P06733_ENOA_HUMAN	ENO1	47.14	3.1325
3	3	P05141_ADT2_HUMAN	SLC25A5	32.83	3.1104
3	3	Q02383_SEMG2_HUMAN	SEMG2	65.41	3.1085
3	3	P18846_ATF1_HUMAN	ATF1	29.21	3.0964
3	3	P08670_VIME_HUMAN	VIM	53.62	3.0747
3	3	Q9H4B7_TBB1_HUMAN	TUBB1	50.29	2.9917
3	3	P04908_H2A1B_HUMAN	HIST1H2AB	14.13	2.9784
3	3	Q13509_TBB3_HUMAN	TUBB3	50.4	2.9386
3	3	P18754_RCC1_HUMAN	RCC1	44.94	2.6984
3	3	Q12905_ILF2_HUMAN	ILF2	43.04	2.5999
3	3	P01876_IGHA1_HUMAN	IGHA1	37.63	2.5787
3	3	Q9Y230_RUVB2_HUMAN	RUVBL2	51.12	2.5563
3	3	P40939_ECHA_HUMAN	HADHA	82.95	2.5555
3	3	Q06830_PRDX1_HUMAN	PRDX1	22.1	2.5457
3	3	Q07065_CKAP4_HUMAN	CKAP4	65.98	2.2344
3	3	Q9Y265_RUVB1_HUMAN	RUVBL1	50.2	2.2137
2	3	P17535_JUND_HUMAN	JUND	35.15	3.7042
2	3	Q9NXF7_DCA16_HUMAN	DCAF16	24.18	3.678
2	3	Q5T0B9_ZN362_HUMAN	ZNF362	45.79	3.3234
2	3	Q8NHW5_RLA0L_HUMAN	RPLP0P6	34.34	3.1873
2	3	P15880_RS2_HUMAN	RPS2	31.3	2.6329
2	3	P62081_RS7_HUMAN	RPS7	22.11	2.0019
2	2	P17544_ATF7_HUMAN	ATF7	52.93	3.9408

2	2	Q9NZI7_UBIP1_HUMAN	UBP1	60.45	3.8354
2	2	P40424_PBX1_HUMAN	PBX1	46.6	3.8171
2	2	Q9BUI4_RPC3_HUMAN	POLR3C	60.57	3.6999
2	2	P83916_CBX1_HUMAN	CBX1	21.4	3.6281
2	2	P61978_HNRPK_HUMAN	HNRNPK	50.94	3.4552
2	2	P62913_RL11_HUMAN	RPL11	20.24	3.4448
2	2	Q13185_CBX3_HUMAN	CBX3	20.8	3.3953
2	2	O43390_HNRPR_HUMAN	HNRNPR	70.9	3.3226
2	2	P04406_G3P_HUMAN	GAPDH	36.03	3.2598
2	2	Q9H2Z4_NKX24_HUMAN	NKX2-4	36.16	3.2302
2	2	O15355_PPM1G_HUMAN	PPM1G	59.23	3.2139
2	2	Q9Y530_OARD1_HUMAN	OARD1	17.01	3.2134
2	2	B7ZW38_HNRC3_HUMAN	HNRNPCL3	32.01	3.0502
2	2	Q7Z6I8_CE024_HUMAN	C5orf24	20.12	3.0351
2	2	P56177_DLX1_HUMAN	DLX1	27.3	2.948
2	2	P10599_THIO_HUMAN	TXN	11.73	2.9448
2	2	P08238_HS90B_HUMAN	HSP90AB1	83.21	2.8932
2	2	Q15233_NONO_HUMAN	NONO	54.2	2.8634
2	2	P78371_TCPB_HUMAN	CCT2	57.45	2.736
2	2	P62263_RS14_HUMAN	RPS14	16.26	2.7058
2	2	Q13952_NFYC_HUMAN	NFYC	50.27	2.7008
2	2	P35244_RFA3_HUMAN	RPA3	13.56	2.6307
2	2	Q71UI9_H2AV_HUMAN	H2AFV	13.5	2.6109
2	2	Q9Y5B9_SP16H_HUMAN	SUPT16H	119.84	2.5449
2	2	Q13867_BLMH_HUMAN	BLMH	52.53	2.535

2	2	P35659_DEK_HUMAN	DEK	42.65	2.5007
2	2	P28370_SMCA1_HUMAN	SMARCA1	122.53	2.4592
2	2	P62249_RS16_HUMAN	RPS16	16.44	2.4534
2	2	P62807_H2B1C_HUMAN	HIST1H2BC	13.9	2.4103
2	2	Q9UQ35_SRRM2_HUMAN	SRRM2	299.44	2.4032
2	2	P62277_RS13_HUMAN	RPS13	17.21	2.3978
2	2	Q02539_H11_HUMAN	HIST1H1A	21.83	2.3776
2	2	Q96A08_H2B1A_HUMAN	HIST1H2BA	14.16	2.3395
2	2	P62269_RS18_HUMAN	RPS18	17.71	2.2306
2	2	P25311_ZA2G_HUMAN	AZGP1	34.24	2.1657
2	2	Q9HCC0_MCCB_HUMAN	MCCC2	61.29	1.9686
1	3	Q2TV78_MST1L_HUMAN	MST1L	79.64	1.4163
1	2	P55209_NP1L1_HUMAN	NAP1L1	45.35	2.5539
1	2	P20226_TBP_HUMAN	TBP	37.67	2.5062
1	2	P01834_IGKC_HUMAN	IGKC	11.6	2.4543
1	2	P32119_PRDX2_HUMAN	PRDX2	21.88	2.3775
1	2	Q9UMX1_SUFU_HUMAN	SUFU	53.91	2.3295
1	1	Q01664_TFAP4_HUMAN	TFAP4	38.7	4.9601
1	1	P35030_TRY3_HUMAN	PRSS3	32.51	4.4898
1	1	Q969G3_SMCE1_HUMAN	SMARCE1	46.62	4.2384
1	1	P07477_TRY1_HUMAN	PRSS1	26.54	4.1884
1	1	P19388_RPAB1_HUMAN	POLR2E	24.54	4.1523
1	1	O75531_BAF_HUMAN	BANF1	10.05	4.1056
1	1	P10515_ODP2_HUMAN	DLAT	68.95	4.0879
1	1	J3KS37_J3KS37_HUMAN	RECQL5	24.89	4.0608

1	1	P61626_LYSC_HUMAN	LYZ	16.53	4.0435
1	1	P35580_MYH10_HUMAN	MYH10	228.86	3.9329
1	1	Q07666_KHDR1_HUMAN	KHDRBS1	48.2	3.8457
1	1	Q14103_HNRPD_HUMAN	HNRNPD	38.41	3.6693
1	1	P52292_IMA1_HUMAN	KPNA2	57.83	3.5634
1	1	P40425_PBX2_HUMAN	PBX2	45.85	3.5308
1	1	P12724_ECP_HUMAN	RNASE3	18.37	3.5157
1	1	P04040_CATA_HUMAN	CAT	59.72	3.4987
1	1	Q9BRT9_SLD5_HUMAN	GINS4	26.03	3.455
1	1	P05387_RLA2_HUMAN	RPLP2	11.66	3.4254
1	1	Q9UGM3_DMBT1_HUMAN	DMBT1	260.57	3.3814
1	1	Q99607_ELF4_HUMAN	ELF4	70.69	3.3063
1	1	P41219_PERI_HUMAN	PRPH	53.62	3.2423
1	1	P31271_HXA13_HUMAN	HOXA13	39.7	3.2304
1	1	P04350_TBB4A_HUMAN	TUBB4A	49.55	3.2212
1	1	B2RPK0_HGB1A_HUMAN	HMGB1P1	24.22	3.22
1	1	P17661_DESM_HUMAN	DES	53.5	3.2016
1	1	Q58FF8_H90B2_HUMAN	HSP90AB2P	44.32	3.1443
1	1	Q9UJU2_LEF1_HUMAN	LEF1	44.17	3.1367
1	1	Q9UQD0_SCN8A_HUMAN	SCN8A	225.13	3.1227
1	1	Q01658_NC2B_HUMAN	DR1	19.43	3.102
1	1	P81877_SSBP2_HUMAN	SSBP2	37.8	3.0627
1	1	Q12873_CHD3_HUMAN	CHD3	226.45	3.053
1	1	P06748_NPM_HUMAN	NPM1	32.55	3.013
1	1	P07199_CENPB_HUMAN	CENPB	65.13	3.003

1	1	Q8WXX5_DNJC9_HUMAN	DNAJC9	29.89	2.9986
1	1	Q8N7H5_PAF1_HUMAN	PAF1	59.94	2.9981
1	1	O60506_HNRPQ_HUMAN	SYNCRIP	69.56	2.998
1	1	P60842_IF4A1_HUMAN	EIF4A1	46.12	2.9945
1	1	Q92922_SMRC1_HUMAN	SMARCC1	122.79	2.9524
1	1	P68371_TBB4B_HUMAN	TUBB4B	49.8	2.9066
1	1	P10909_CLUS_HUMAN	CLU	52.46	2.8497
1	1	O15318_RPC7_HUMAN	POLR3G	25.9	2.8476
1	1	Q14566_MCM6_HUMAN	MCM6	92.83	2.8081
1	1	Q03060_CREM_HUMAN	CREM	38.92	2.7959
1	1	P52434_RPAB3_HUMAN	POLR2H	17.13	2.7668
1	1	P31689_DNJA1_HUMAN	DNAJA1	44.84	2.7075
1	1	P52907_CAZA1_HUMAN	CAPZA1	32.9	2.7002
1	1	P25705_ATPA_HUMAN	ATP5A1	59.71	2.6172
1	1	P06576_ATPB_HUMAN	ATP5B	56.52	2.5974
1	1	P50402_EMD_HUMAN	EMD	28.98	2.5389
1	1	P33992_MCM5_HUMAN	MCM5	82.23	2.5236
1	1	P49411_EFTU_HUMAN	TUFM	49.51	2.4903
1	1	Q6ZVX7_FBX50_HUMAN	NCCRP1	30.83	2.4509
1	1	P01857_IGHG1_HUMAN	IGHG1	36.08	2.4341
1	1	P49916_DNLI3_HUMAN	LIG3	112.83	2.411
1	1	Q15431_SYCP1_HUMAN	SYCP1	114.12	2.404
1	1	Q07687_DLX2_HUMAN	DLX2	34.22	2.349
1	1	P02788_TRFL_HUMAN	LTF	78.13	2.3235
1	1	P17480_UBF1_HUMAN	UBTF	89.35	2.321

1	1	P46783_RS10_HUMAN	RPS10	18.89	2.3067
1	1	P14649_MYL6B_HUMAN	MYL6B	22.75	2.293
1	1	Q15393_SF3B3_HUMAN	SF3B3	135.49	2.2884
1	1	O95677_EYA4_HUMAN	EYA4	69.46	2.2796
1	1	P00491_PNPH_HUMAN	PNP	32.1	2.2743
1	1	P62851_RS25_HUMAN	RPS25	13.73	2.2593
1	1	Q9UHN1_DPOG2_HUMAN	POLG2	54.88	2.246
1	1	P68871_HBB_HUMAN	HBB	15.99	2.2043
1	1	Q8N8E3_CE112_HUMAN	CEP112	112.68	2.1741
1	1	Q14978_NOLC1_HUMAN	NOLC1	73.56	2.1625
1	1	Q8IVW6_ARI3B_HUMAN	ARID3B	60.6	2.1484
1	1	P28332_ADH6_HUMAN	ADH6	39.06	2.1302
1	1	P17482_HXB9_HUMAN	HOXB9	28.04	2.1108
1	1	O75367_H2AY_HUMAN	H2AFY	39.59	2.1006
1	1	P13639_EF2_HUMAN	EEF2	95.28	2.0792
1	1	Q7L5L3_GDPD3_HUMAN	GDPD3	36.57	2.0049
1	1	P62244_RS15A_HUMAN	RPS15A	14.83	2.0004
1	1	P45880_VDAC2_HUMAN	VDAC2	31.55	1.9732
1	1	P62826_RAN_HUMAN	RAN	24.41	1.9251
1	1	P22670_RFX1_HUMAN	RFX1	104.69	1.924
1	1	Q9C030_TRIM6_HUMAN	TRIM6	56.36	1.9152
1	1	P22314_UBA1_HUMAN	UBA1	117.77	1.9071
1	1	O43175_SERA_HUMAN	PHGDH	56.61	1.8929
1	1	P69905_HBA_HUMAN	HBA1	15.25	1.8706
1	1	Q13162_PRDX4_HUMAN	PRDX4	30.52	1.8574

1	1	Q9NVU0_RPC5_HUMAN	POLR3E	79.85	1.855
1	1	O43248_HXC11_HUMAN	HOXC11	33.73	1.8328
1	1	H0YCG2_H0YCG2_HUMAN	LAMP2	28.25	1.8086

Supplemental Table S2. Summary of tandem affinity purification and mass spectrometry of mutant tPARP1 (mtPARP1), mutant TARG1 (mTARG1), mutant PARP1 (mPARP1) and mutant PARG (mPARG).

mtPARP1			mPARP1			mTARG1			mPARG		
Unique	Total	Gene Symbol	Unique	Total	Gene Symbol	Unique	Total	Gene Symbol	Unique	Total	Gene Symbol
109	377	PARP1	108	480	PARP1	94	443	PARP1	104	786	PARG
54	83	XRCC6	74	121	HLTF	83	96	ACACA	66	95	PARP1
53	86	XRCC5	70	124	SUPT16H	60	169	SUPT16H	32	85	PCNA
19	19	DDB1	57	61	PRKDC	56	217	XRCC5	40	74	HSPA8
16	16	HSPA8	53	165	XRCC5	50	179	XRCC6	44	61	HSPD1
15	17	HSPA5	50	123	XRCC6	49	66	HLTF	41	50	XRCC6
13	13	HSPA9	44	58	HNRNPM	40	43	PC	40	49	XRCC5
12	12	HSPA1A	43	49	CHD1L	34	41	LIG3	36	38	PRKDC
11	12	HLTF	40	48	DHX9	33	98	RPA1	35	37	SUPT16H
10	12	HIST1H4A	36	53	LIG3	32	80	SSRP1	23	37	HSPA1A
10	10	RPS3	34	70	RPA1	31	46	MCCC1	27	36	HSPA5
9	11	HSPA1L	34	42	DHX30	26	366	PCNA	23	28	TUBA1A
8	9	TUBB2A	33	36	HDLBP	26	32	NCL	21	28	RAE1
7	7	POLR1C	32	45	HNRNPU	26	28	PCCA	23	26	HSPA9
7	7	TFAM	30	60	NCL	25	56	MCCC2	22	26	TUBB2A
7	7	RPA1	30	54	SSRP1	24	378	OARD1	22	26	PPP2R1A
6	7	MCCC1	29	49	TMPO	24	25	CHD1L	19	26	PRDX1
6	6	TUBA1A	29	32	CKAP5	23	26	PARP2	11	26	HSPA1L
6	6	POLR3A	28	33	YTHDC2	22	27	THRAP3	22	25	HSPH1
5	6	HIST1H2BB	28	31	GTPBP4	22	24	XPC	21	25	EEF2
5	5	SMARCA5	27	48	RPL4	22	24	WRN	19	22	PKM
5	5	SEMG1	26	336	OARD1	21	22	CKAP4	19	21	HSPA4
5	5	ANXA2	26	72	RPS4X	21	21	DHX9	19	21	RPS3
5	5	CHAF1B	26	35	DDX17	20	22	PCCB	19	21	DDB1
4	7	HIST3H3	26	31	SMARCAL1	19	22	RFC1	16	20	USP9X
4	5	SSBP1	26	29	HADHA	18	23	BCLAF1	18	19	AHCYL1
4	5	TTF2	25	52	RPL3	17	21	IMPDH2	18	19	SSRP1
4	4	CREB1	25	47	SERBP1	16	29	RPS4X	17	19	DNAJC7
4	4	MCM7	25	44	RPS3A	16	18	XRCC1	18	18	PRMT5
4	4	UBA52	23	69	RPS3	15	27	RPS3	17	18	HLTF
4	4	EEF1A1	23	35	TOP1	15	24	DBT	16	18	PPP2R2A
4	4	HSPA2	23	32	PARP2	15	24	RPL3	17	17	LIG3
4	4	POLR3B	23	31	RCC2	14	15	RPL7	13	17	EEF1A1
4	4	HIST1H2AA	23	30	DDX21	14	14	RFC4	15	16	HSP90AA1

4	4	RPA2	22	45	EEF1A1	14	14	DDX21	15	16	STIP1
4	4	POLR3F	22	28	AIFM1	14	14	HNRNPM	14	16	CCT2
3	4	HSPD1	22	26	RFC1	13	25	NPM1	15	15	RPA1
3	4	HOXA10	22	26	SYNCRIP	13	14	PNKP	15	15	CHFR
3	4	XRCC4	22	24	TOP2A	13	14	RPL4	15	15	TUFM
3	4	ATF2	22	22	IQGAP1	13	13	ILF2	14	15	CHD1L
3	4	HNRNPC	21	33	DDX5	13	13	RFC2	14	14	TCP1
3	3	TFCP2	21	31	DDX3Y	13	13	RPS9	14	14	STRAP
3	3	JUN	21	23	RTCB	12	14	KPNA2	13	14	TMPO
3	3	HNRNPH1	21	22	UBTF	12	14	HSPA8	13	14	HNRNPU
3	3	RBBP4	20	23	DHX15	12	13	FBXO18	13	13	CAD
3	3	ZNF148	20	21	CDC5L	12	12	SYNCRIP	11	13	STUB1
3	3	CDSN	20	21	MYBBP1A	12	12	DDX5	11	13	HSPA4L
3	3	ENO1	19	22	DDX1	11	68	RPA2	11	13	HSP90AB3P
3	3	SLC25A5	19	22	ATAD3A	11	12	RFC5	12	12	CTPS1
3	3	SEMG2	19	21	ABCF1	11	11	AMOT	12	12	USP7
3	3	ATF1	19	20	WRN	10	13	RPS2	12	12	RPS4X
3	3	VIM	18	39	RPS9	10	13	RPS6	11	12	TOPBP1
3	3	TUBB1	18	25	HSPA8	10	12	RPL7A	9	12	HIST1H4A
3	3	HIST1H2AB	18	21	TSR1	10	10	HSPA1A	9	12	GRWD1
3	3	TUBB3	18	21	POP1	10	10	IGF2BP1	11	11	PARP2
3	3	RCC1	18	19	ZC3HAV1	9	26	HIST1H4A	9	11	HSP90AB1
3	3	ILF2	17	34	RPS6	9	23	RPA3	10	10	ACTA2
3	3	IGHA1	17	24	RPL7	9	15	RPL13	9	10	NUP155
3	3	RUVBL2	17	21	ILF3	9	13	RPS3A	6	10	NPM1
3	3	HADHA	17	18	DHX36	9	11	HSPA1L	5	10	TMPO
3	3	PRDX1	16	44	NPM1	9	11	RPS18	9	9	PPP6R3
3	3	CKAP4	16	36	RPL23	9	11	NOLC1	9	9	PCBP1
3	3	RUVBL1	16	26	XRCC1	9	10	TUBB2A	9	9	PAICS
			16	19	STAU1	9	10	CSNK2A1	9	9	XRCC1
			16	18	GNL3	9	10	RPL8	9	9	RPS9
			16	18	IRS4	9	9	MYBBP1A	8	9	HBB
			15	22	RPS19	9	9	RPL6	8	9	PCMT1
			15	17	MAP4	8	10	RPS15A	8	9	RCC2
			15	16	DHX29	8	9	RPL9	8	9	BUB3
			15	16	PTCD3	8	9	CETN2	8	9	HSP90AB2P
			14	28	RPS18	8	9	TERF1	7	9	PLS3
			14	28	PTBP1	8	9	RPS7	5	9	TUBB3
			14	25	RPL7A	8	9	POLR3A	8	8	MAPK1
			14	20	PRPF19	8	9	HSPA9	8	8	AIFM1
			14	19	ILF2	8	8	CHTF18	8	8	EIF4A1
			14	19	RPL13	8	8	TUBA1A	8	8	FASN
			14	17	G3BP1	8	8	SMARCAL1	8	8	PRDX2

	14	16	IGF2BP1	8	8	RFC3	8	8	MSH2
	14	15	EXOSC10	8	8	ILF3	8	8	GART
	14	15	ASCC3	8	8	RPL15	8	8	RPL4
	13	28	TMPO	8	8	RPL26L1	7	8	CAPZA1
	13	18	RPL23A	7	10	YBX1	7	8	ENO1
	13	18	RPL6	7	8	SFPQ	7	8	RPS3A
	13	17	SRPK1	7	7	APTX	7	8	HSPA2
	13	16	RPL26L1	7	7	TXNRD1	7	8	CDK5
	13	15	LEMD2	7	7	GNL3	6	8	GPX4
	13	14	DHX57	7	7	RPS16	5	8	HIST1H2BB
	13	14	PNKP	7	7	RPS17L	5	8	RPA2
	13	14	HADHB	6	198		4	8	TUBB1
	13	13	GNL2	6	24	HIST1H2BB	7	7	VPS35
	13	13	CSDE1	6	8	TDP1	7	7	UBA1
	13	13	RRP1B	6	7	SSBP1	7	7	HNRNPH1
	12	24	RPA2	6	7	DDX17	7	7	BAG5
	12	23	RPLP0P6	6	7	RPS8	7	7	EEF1G
	12	17	HNRNPK	6	7	RPL31	7	7	CCT3
	12	15	POLRMT	6	7	FEN1	7	7	DDX1
	12	15	RPS7	6	6	CDC73	7	7	CPNE3
	12	14	MRPS27	6	6	RAD23B	7	7	MAPK3
	12	13	MRPS22	6	6	RPS19	6	7	LRRC47
	12	12	CKAP4	6	6	RPS13	6	7	RPS13
	12	12	EMD	6	6	PRMT1	6	7	PRPSAP2
	12	12	CCDC82	6	6	TOP1	6	7	AHCY
	12	12	XRN1	5	8	RPL17	6	7	GMPS
	11	25	RPS16	5	7	RPS14	6	7	GARS
	11	24	RPS14	5	7	RPL23	6	7	RPS18
	11	24	RPS2	5	7	HIST3H3	5	7	ACTB
	11	22	RPS13	5	6	RPLP0	5	7	YWHAQ
	11	20	RPS17L	5	6	FEM1B	5	7	TUBB
	11	20	RPL18A	5	6	NAT10	5	7	RPS2
	11	16	TUBB2A	5	6	POLDIP3	6	6	HNRNPK
	11	16	HSPA1L	5	6	HNRNPAB	6	6	IMPDH2
	11	15	HNRNPD	5	6	RPL23A	6	6	PPP2CA
	11	15	HSPA1A	5	6	PRPF19	6	6	JMJD4
	11	15	LMNA	5	5	RPLP2	6	6	DNAJA1
	11	15	RPS15A	5	5	CSNK2A2	6	6	RUVBL1
	11	14	RFC2	5	5	CAPRIN1	6	6	RNH1
	11	14	UPF3B	5	5	TIMELESS	6	6	CACYBP
	11	14	CAPRIN1	5	5	GTF2I	6	6	AHCYL2
	11	14	ABCF2	5	5	RPL18	6	6	IRS4
	11	13	IMPDH2	5	5	CUL2	6	6	MCMBP

	11	13	EIF5B	5	5	SLC25A4	6	6	NCL
	11	13	HNRNPR	5	5	KIAA0101	6	6	RPS16
	11	12	NOP58	5	5	DHX15	6	6	KEAP1
	11	12	DRG1	5	5	CDC5L	6	6	RPL7
	11	12	EIF2AK2	5	5	RPS11	6	6	RPL3
	11	12	STRAP	5	5	RPLP0P6	5	6	PPM1G
	11	12	RPL5	5	5	RPL35	5	6	DHX15
	11	11	HSPA9	5	5	RPL10A	5	6	CHEK1
	11	11	MTDH	4	6	RPL13AP3	5	6	CFL1
	11	11	MRPS35	4	5	RPS15	5	6	PLCG1
	11	11	LIG4	4	5	RPL24	3	6	DNAJA2
	11	11	CKAP2	4	5	DDB1	5	5	RPLP0
	11	11	TFB1M	4	4	HNRNPU	5	5	HSD17B10
	10	18	NHEJ1	4	4	POLR1C	5	5	FKBP5
	10	15	DAP3	4	4	HNRNPR	5	5	WDR77
	10	14	NSUN2	4	4	HNRNPA1	5	5	NAGK
	10	13	PRRC2A	4	4	HNRNPA1L2	5	5	HAT1
	10	12	TUBA1A	4	4	EIF2S3L	5	5	TUBB4A
	10	12	RFC5	4	4	SKP1	5	5	TXN
	10	12	NONO	4	4	PURA	5	5	KPNA2
	10	11	SART1	4	4	RPL12	5	5	THNSL1
	10	11	PIP5K1A	4	4	RNF187	5	5	RPLP0P6
	10	11	ZC3H15	4	4	APLF	5	5	SLC25A4
	10	11	DEK	4	4	TRIM28	5	5	MSH3
	10	11	LARP1	4	4	HIST1H1C	5	5	MCCC1
	10	11	KIF2A	4	4	MRPS27	5	5	AASDHPPT
	10	11	DDX41	4	4	LYAR	5	5	PRPS1
	10	11	EPRS	4	4	HP1BP3	5	5	FLAD1
	10	10	NAT10	4	4	RPS23	5	5	RPL10A
	10	10	SRP68	4	4	IRS4	5	5	EIF5A
	10	10	TDP1	4	4	RPL30	5	5	RPS7
	10	10	LRRC47	4	4	PPM1G	5	5	DHX9
	10	10	AP1B1	4	4	RPS25	5	5	BAG2
	10	10	MINA	4	4	POLB	5	5	DDX17
	10	10	MOGS	4	4	ZC3HAV1	5	5	TTC4
	10	10	LTV1	4	4	CUL1	5	5	RPS11
	10	10	CDC73	3	8	RPS24	4	5	CDK4
	10	10	LRPPRC	3	6	HIST1H2AA	4	5	HSPBP1
	9	36	HIST1H4A	3	4	RBM39	4	5	GNB1L
	9	22	RPL31	3	4	POLR3C	4	4	YBX1
	9	16	RPL18	3	4	RPL19	4	4	PAXIP1
	9	15	YBX1	3	4	DDX47	4	4	SF3B3
	9	13	RPS15	3	4	UBA52	4	4	HNRNPF

	9	12	AP2B1	3	4	RPL11	4	4	RUVBL2
	9	12	DHX37	3	4	POLR3B	4	4	PFAS
	9	12	EIF2S2	3	4	FUS	4	4	EMD
	9	11	HSD17B4	3	4	RPL27A	4	4	CAPZB
	9	11	GRWD1	3	4	RPL10	4	4	RPS8
	9	11	GNL3L	3	4	HNRNPA2B1	4	4	ILF3
	9	11	ARHGEF2	3	3	RPL22	4	4	RPL23A
	9	11	AP2M1	3	3	DHX30	4	4	CCT4
	9	10	HNRNPF	3	3	GRWD1	4	4	HBA1
	9	10	RPL10A	3	3	DDX3X	4	4	SLC25A13
	9	10	RPL15	3	3	DDX50	4	4	MCM5
	9	9	KPNA2	3	3	NOP2	4	4	AHSA1
	9	9	IARS	3	3	PSIP1	4	4	HPRT1
	9	9	ASPH	3	3	SLC25A5	4	4	MIB2
	9	9	RARS	3	3	GTPBP4	4	4	RPS6
	9	9	EEF2	3	3	HNRNPA0	4	4	PPP5C
	9	9	HSPD1	3	3	NONO	4	4	CRKL
	9	9	DNMT1	3	3	RRP1B	4	4	MCM3
	9	9	EIF2S1	3	3	MTDH	4	4	WDR48
	9	9	PRPF31	3	3	PNO1	4	4	USP11
	9	9	DKC1	3	3	RPL14	4	4	IPO4
	9	9	DDX18	3	3	DKC1	4	4	THOC3
	9	9	PRDX1	3	3	HSPA2	4	4	WRNIP1
	8	14	HNRNPH1	3	3	IPO9	4	4	MCCC2
	8	14	PA2G4	3	3	HSPA5	4	4	PRPF19
	8	12	DNAJA2	3	3	ERH	4	4	DDX5
	8	11	HNRNPAB	3	3	POLR3E	4	4	RPS19
	8	11	KRR1	3	3	RPL34	4	4	RPL13
	8	10	SMC3	3	3	TUBB3	4	4	GRB2
	8	10	APTX	3	3	RPS26P11	3	4	TUBB6
	8	10	RPL8	3	3	RPL18A	3	4	SERBP1
	8	9	SARS2	3	3	CHFR	3	4	DDX3X
	8	9	TRMT6	3	3	RPL28	3	4	TARDBP
	8	9	DNAJC2	3	3	RPL13A	3	4	PCBP2
	8	9	FBXO18	3	3	RPL5	3	4	PRPSAP1
	8	9	TIMELESS				3	4	MPO
	8	9	HMMR				2	4	CAPZA2
	8	9	NUSAP1				2	4	HSP90AA2
	8	9	RNMTL1				3	3	RCN2
	8	9	RPL28				3	3	FKBP8
	8	8	DNAJA1				3	3	FBXO22
	8	8	SND1				3	3	RPL18
	8	8	POLB				3	3	IGF2BP1

	8	8	TUFM		3	3	ILF2
	8	8	NOP56		3	3	MRPS27
	8	8	HSPA5		3	3	RPS14
	8	8	MARK2		3	3	RPL12
	8	8	MSH3		3	3	CSK
	8	8	NOP14		3	3	HNRNPM
	8	8	AP2A1		3	3	DHPS
	8	8	BYSL		3	3	LRRC1
	8	8	POLDIP3		3	3	RPL11
	8	8	SRPR		3	3	OLA1
	8	8	DDX50		3	3	KPNB1
	8	8	BLM		3	3	HSP90AA5P
	8	8	RPS11		3	3	SLC25A5
	7	28	RPS24		3	3	USP47
	7	24	RPL24		3	3	RPL31
	7	16	RPL17		3	3	RPS24
	7	16	RPL19		3	3	CPOX
	7	15	RPL10		3	3	PEX7
	7	12	RPS8		3	3	PMPCA
	7	10	U2AF2		3	3	CCT8
	7	10	TRIM28		3	3	RPS20
	7	10	ZNF598		3	3	MCM7
	7	10	RPLP0		3	3	HIST1H2AA
	7	10	RPL21		3	3	LTF
	7	10	RPS10P5		3	3	KPNA1
	7	10	SLC25A4		3	3	SYNCRIP
	7	9	CHFR		3	3	MAPK14
	7	9	FAM98A		3	3	RPL23
	7	9	RRBP1		3	3	ATP5B
	7	9	RBM39		3	3	ATP1A1
	7	9	DDX49		3	3	IARS
	7	9	RECQL		3	3	BPIFB1
	7	9	MRPS5		3	3	PPIA
	7	9	RPL13A		3	3	RPS25
	7	8	GTPBP1		3	3	LCMT2
	7	8	TRIM25		3	3	RPL17
	7	8	PARG		3	3	PHGDH
	7	8	EIF2D		3	3	ELP2
	7	8	RPL9		3	3	RBBP4
	7	8	USP10		3	3	SLC25A3
	7	8	ACTA2		3	3	DCAF16
	7	8	PRPF3		3	3	CYFIP1
	7	8	CLPB		3	3	FTO

	7	8	GMDS		3	3	RPL6
	7	8	RPL32		3	3	TBCB
	7	8	RSBN1L		3	3	RPL7A
	7	8	RPL35		3	3	EPRS
	7	7	SLIT2		3	3	RPL27
	7	7	ZNF281		3	3	SMARCAL1
	7	7	LEMD3		3	3	RPL13A
	7	7	MRPS31		3	3	PABPC1
	7	7	PLRG1		3	3	HIST3H3
	7	7	RFC4		3	3	WRN
	7	7	G3BP2		3	3	ATAD3A
	7	7	GTSE1		3	3	RPL35A
	7	7	MAPK1				
	7	7	HSPA14				
	7	7	NSUN5				
	7	7	NOA1				
	7	7	UHRF1				
	7	7	DDX54				
	7	7	TBL2				
	7	7	BRIX1				
	7	7	DDX47				
	7	7	DARS				
	7	7	HP1BP3				
	7	7	PRC1				
	7	7	NOC4L				
	7	7	EIF2S3L				
	7	7	XRCC4				
	7	7	RSL1D1				
	7	7	PES1				
	6	42	HIST1H2BB				
	6	14	BANF1				
	6	14	RPA3				
	6	11	DDX3X				
	6	9	HNRNPA0				
	6	9	HNRNPA1L2				
	6	9	RPL12				
	6	9	RPL36A				
	6	8	RPL14				
	6	7	FAM98B				
	6	7	SUN2				
	6	7	DDX55				
	6	7	MSH2				
	6	7	SMC1A				

	6 7 SRPK2			
	6 7 MTHFD1L			
	6 7 MRPS18B			
	6 7 EIF2A			
	6 7 MTPAP			
	6 7 SNW1			
	6 7 CC2D1A			
	6 7 MRPS34			
	6 6 CHTF18			
	6 6 PEO1			
	6 6 SSB			
	6 6 UTP14A			
	6 6 MAP7D1			
	6 6 NOP2			
	6 6 CLASP1			
	6 6 PLK1			
	6 6 RPP30			
	6 6 PRPF4			
	6 6 NEMF			
	6 6 CNP			
	6 6 LYAR			
	6 6 VRK1			
	6 6 KIFC1			
	6 6 SFPQ			
	6 6 SLC25A13			
	6 6 TPX2			
	6 6 HNRNPA2B1			
	6 6 PABPC1			
	6 6 SKIV2L2			
	6 6 RPS5			
	5 11 RPL11			
	5 10 RPLP2			
	5 10 HNRNPH2			
	5 10 RPL13AP3			
	5 8 RPS23			
	5 7 RPL30			
	5 7 BOP1			
	5 7 PWP1			
	5 7 HIST1H1C			
	5 7 RPS25			
	5 7 RPL27			
	5 6 DDX52			
	5 6 TRIP12			

	5	6	IGF2BP2		
	5	6	ADAR		
	5	6	MARK3		
	5	6	YTHDF2		
	5	6	DDX24		
	5	6	PPM1G		
	5	6	AP2A2		
	5	6	KRI1		
	5	6	DDX28		
	5	6	CDKAL1		
	5	6	MARS		
	5	6	SDAD1		
	5	6	NOM1		
	5	6	XRN2		
	5	6	LRRC59		
	5	6	RPL36		
	5	6	FARSA		
	5	6	RPL38		
	5	6	RPL35A		
	5	5	RPSA		
	5	5	DDX6		
	5	5	CSNK1D		
	5	5	BMS1		
	5	5	TCEB3		
	5	5	FXR1		
	5	5	VRK2		
	5	5	NIFK		
	5	5	HNRNPA3		
	5	5	GRSF1		
	5	5	MCM5		
	5	5	THRAP3		
	5	5	QARS		
	5	5	NCAPG2		
	5	5	CUL1		
	5	5	SNRPA1		
	5	5	HMCES		
	5	5	RBMX		
	5	5	USP7		
	5	5	GPATCH4		
	5	5	DDX56		
	5	5	IGHMBP2		
	5	5	RRS1		
	5	5	RFC3		

	5	5	TRMT2A		
	5	5	SRSF5		
	5	5	CMAS		
	5	5	H1FX		
	5	5	MRPS7		
	5	5	FARSB		
	5	5	FUS		
	5	5	SMC2		
	5	5	ASCC2		
	5	5	AURKA		
	5	5	ABCD3		
	5	5	DIMT1		
	5	5	FBL		
	5	5	DNAJC9		
	5	5	TOP1MT		
	5	5	BCLAF1		
	4	12	RPS26P11		
	4	11	RPS10		
	4	8	ACTB		
	4	7	UBA52		
	4	7	RPL10L		
	4	6	MRPS23		
	4	6	TECR		
	4	6	RPL34		
	4	5	EWSR1		
	4	5	WDR6		
	4	5	KIF23		
	4	5	RNH1		
	4	5	APLF		
	4	5	SRP72		
	4	5	HNRNPA1		
	4	5	MRPS9		
	4	5	MUS81		
	4	5	PSIP1		
	4	5	ESF1		
	4	5	ZNF768		
	4	5	SNRPD3		
	4	5	WDR3		
	4	5	RBM14		
	4	5	SRSF3		
	4	5	MPG		
	4	5	HELLS		
	4	4	LSM14B		

	4	4	H2AFY		
	4	4	FTSJ3		
	4	4	C4orf27		
	4	4	TBL3		
	4	4	C14orf166		
	4	4	TCP1		
	4	4	RBM28		
	4	4	GTPBP10		
	4	4	ESPL1		
	4	4	TMEM214		
	4	4	TOP2B		
	4	4	TXNRD1		
	4	4	TUBB		
	4	4	BUB3		
	4	4	RRP7A		
	4	4	RTCA		
	4	4	HSPA2		
	4	4	CFL1		
	4	4	MRPS10		
	4	4	SMU1		
	4	4	RPN1		
	4	4	KIF14		
	4	4	RPL22		
	4	4	WDR12		
	4	4	VRK3		
	4	4	SGPL1		
	4	4	PCBP2		
	4	4	IGF2BP3		
	4	4	SMCHD1		
	4	4	SLC25A3		
	4	4	MYH9		
	4	4	PRMT1		
	4	4	KIF2C		
	4	4	AP3D1		
	4	4	LLGL1		
	4	4	PTCD1		
	4	4	SLC25A11		
	4	4	DNAJA3		
	4	4	LRWD1		
	4	4	PTPLAD1		
	4	4	SMARCA5		
	4	4	CDC20		
	4	4	TRMT10C		

	4	4	DDX10		
	4	4	THOC2		
	4	4	KIN		
	4	4	EIF4G1		
	4	4	NXF1		
	4	4	TFAM		
	4	4	SPATA5L1		
	4	4	NOB1		
	4	4	RPP38		
	4	4	AURKB		
	4	4	TOE1		
	4	4	HNRNPLL		
	4	4	CLASP2		
	4	4	RBBP4		
	4	4	CPT1A		
	4	4	SRSF4		
	4	4	HIST3H3		
	3	9	HIST1H1A		
	3	8	HIST1H2AA		
	3	7	SSBP1		
	3	6	RPS27L		
	3	6	SLC25A5		
	3	6	RBM42		
	3	6	KARS		
	3	5	YBX3		
	3	5	EIF3D		
	3	5	RPL3L		
	3	5	RPS12		
	3	4	HBS1L		
	3	4	H2AFY2		
	3	4	GTF3C2		
	3	4	RIOK2		
	3	4	TMA16		
	3	4	RPS20		
	3	4	SPATS2L		
	3	4	ZC3H7B		
	3	4	TARS2		
	3	4	CCNB1		
	3	4	EIF4A1		
	3	4	MATR3		
	3	4	AIMP2		
	3	4	MCAT		
	3	4	CAMSAP3		

	3	4	RPL27A		
	3	4	PPAN		
	3	4	CEBPZ		
	3	4	RPS28		
	3	4	CBX4		
	3	4	UPF2		
	3	4	MORF4L1		
	3	4	SRP14		
	3	4	SNRPB		
	3	4	HIST1H2BA		
	3	3	SRPRB		
	3	3	ALKBH5		
	3	3	PDS5A		
	3	3	TARDBP		
	3	3	DNA2		
	3	3	PCBP1		
	3	3	RRP1		
	3	3	EFTUD2		
	3	3	CIRBP		
	3	3	NVL		
	3	3	LUC7L2		
	3	3	MCM3		
	3	3	HERC5		
	3	3	FAM120A		
	3	3	NOP16		
	3	3	DPM1		
	3	3	LARS		
	3	3	MPHOSPH10		
	3	3	RRP12		
	3	3	NACA		
	3	3	TXLNA		
	3	3	SLC16A1		
	3	3	PKN3		
	3	3	USP39		
	3	3	PPIL2		
	3	3	SF3B2		
	3	3	ARHGAP19		
	3	3	CCDC86		
	3	3	TFCP2		
	3	3	SLC25A1		
	3	3	EIF5A		
	3	3	HIRIP3		
	3	3	CAMK2D		

	3	3	SNRNP200			
	3	3	ATAD3B			
	3	3	METAP2			
	3	3	PPP1CA			
	3	3	CWF19L1			
	3	3	TRIM26			
	3	3	STAU2			
	3	3	HS2ST1			
	3	3	GTF2B			
	3	3	SRBD1			
	3	3	YME1L1			
	3	3	GRK6			
	3	3	YTHDF1			
	3	3	RSBN1			
	3	3	U2SURP			
	3	3	GPAM			
	3	3	GTPBP6			
	3	3	PRPSAP1			
	3	3	MAP7D2			
	3	3	RANBP6			
	3	3	MMTAG2			
	3	3	PHF6			
	3	3	ASF1A			
	3	3	EZR			
	3	3	DLG3			
	3	3	TOP3A			
	3	3	PURA			
	3	3	MRPS26			
	3	3	EPB41L5			
	3	3	SMC4			
	3	3	ASTE1			
	3	3	TCF25			
	3	3	ZRANB2			
	3	3	SMC5			
	3	3	EXOSC7			
	3	3	KCTD12			
	3	3	KIAA0391			
	3	3	LUZP1			
	3	3	MCM10			
	3	3	KIF4A			
	3	3	NKRF			
	3	3	HEATR3			
	3	3	PAF1			

	3	3	RCC1		
	3	3	BUD13		
	3	3	EBNA1BP2		
	3	3	SRSF7		
	3	3	PRPF8		
	3	3	KHDRBS1		
	3	3	LARP7		
	3	3	EEF1G		
	3	3	MAP4K4		
	3	3	LARP1B		

Supplemental Table S3. Primer sets for RT-qPCR.

Target	Primers
tRNA-Ala	F: 5'-GGGGGTGTAGCTCAGTGG-3'
	R: 5'-TGGTGGAGGTGTCGGGGATC-3'
tRNA-Gly	F: 5'-GCGTTGGTGGTATAGTGGT-3'
	R: 5'-TGGTGCGTTGGCCGGGAATCGA-3'
tRNA-His	F: 5'-GGCCGTGATCGTATAGTG-3'
	R: 5'-CGAACCGAGGTTGCTGCGG-3'
tRNA-Pro	F: 5'-GGCTCGTTGGTCTAG-3'
	R: 5'-TGGGGGCTCGTCCGGGATTTG-3'
tRNA-Leu	F: 5'-GAGGACAACGGGGACAGTAA-3'
	R: 5'-TCCACCAGAAAAACTCCAGC-3'
U6 snRNA	F: 5'-GCTTCGGCAGCACATATACTA-3'
	R: 5'-CGAATTTGCGTGTCATCCTTG-3'
5S rRNA	F: 5'-TACGGCCATACCACCCTGAAC-3'
	R: 5'-CGGTCTCCCATCCAAGTACTAACC-3'
IFN-β	F: 5'-AGGACAGGATGAACTTTGAC-3'
	R: 5'-TGATAGACATTAGCCAGGAG-3'
GAPDH	F: 5'-GCACCACCAACTGCTTA-3'
	R: 5'-AGTAGAGGCAGGGATGA-3'
